# Roles of inflammatory cell infiltrate in periprosthetic osteolysis

**DOI:** 10.3389/fimmu.2023.1310262

**Published:** 2023-12-01

**Authors:** Isidora Panez-Toro, Dominique Heymann, François Gouin, Jérôme Amiaud, Marie-Françoise Heymann, Luis A. Córdova

**Affiliations:** ^1^ Department of Oral and Maxillofacial Surgery, Faculty of Dentistry, University of Chile, Independencia, Santiago, Chile; ^2^ Nantes Université, Centre National de Recherche Scientifique (CNRS), UMR6286, US2B, Nantes, France; ^3^ Institut de Cancérologie de l’Ouest, Tumor Heterogeneity and Precision Medicine Laboratory, Saint-Herblain, France; ^4^ Nantes Université, Laboratory of Histology and Embryology, Medical School, Nantes, France; ^5^ The University of Sheffield, Dept of Oncology and Metabolism, Sheffield, United Kingdom; ^6^ Department of Surgical Oncology, Centre Léon Bérard, Lyon, France; ^7^ IMPACT, Center of Interventional Medicine for Precision and Advanced Cellular Therapy, Santiago, Chile; ^8^ Oral and Maxillofacial Surgery, Clínica MEDS, Santiago, Chile

**Keywords:** total joint replacement, periprosthetic osteolysis, aseptic loosening, osteoimmunology, macrophage, inflammation, osteoclast, innate immune system

## Abstract

Classically, particle-induced periprosthetic osteolysis at the implant–bone interface has explained the aseptic loosening of joint replacement. This response is preceded by triggering both the innate and acquired immune response with subsequent activation of osteoclasts, the bone-resorbing cells. Although particle-induced periprosthetic osteolysis has been considered a foreign body chronic inflammation mediated by myelomonocytic-derived cells, current reports describe wide heterogeneous inflammatory cells infiltrating the periprosthetic tissues. This review aims to discuss the role of those non-myelomonocytic cells in periprosthetic tissues exposed to wear particles by showing original data. Specifically, we discuss the role of T cells (CD3^+^, CD4^+^, and CD8^+^) and B cells (CD20^+^) coexisting with CD68^+^/TRAP^−^ multinucleated giant cells associated with both polyethylene and metallic particles infiltrating retrieved periprosthetic membranes. This review contributes valuable insight to support the complex cell and molecular mechanisms behind the aseptic loosening theories of orthopedic implants.

## 1 Introduction

Osteoarthritis is an inflammatory disease contributing to the degenerative process of the joint (1). As a treatment, total joint arthroplasty is widely used in orthopedics as a solution due to its cost-effectiveness and success after osteoarthritis (2–4). However, it has been shown that some biomaterials in orthopedic implants, such as polyethylene, metallic alloys, and ceramics, produce wear debris over time by several abrasive and/or corrosive mechanisms (4–6), especially in younger patients (4). This wear over time induces periprosthetic osteolysis, leading to aseptic loosening of the implant (2, 4, 7–15). Newer materials are now available that produce fewer wear particles (16), but this does not eliminate the current clinical issue in which patients present aseptic loosening of their implants.

Usually, following a total joint arthroplasty, a protective homeostatic response is initiated in the surrounding tissues, which eliminates damaged tissues and tries to eliminate foreign non-biological materials to facilitate tissue adaptation (17). This response starts with a first phase of inflammation (18) to achieve scar tissue formation (19), where fibrin degradation fragments enhance the release of transforming growth factor-beta (TGF-β), stimulating the fibroblast migration to the injury site to deposit extracellular matrix (ECM) and restore the initial tissue feature. Meanwhile, macrophages release vascular endothelial cell growth factor (VEGF), promoting new blood vessel formation (19). Likewise, the implanted biomaterial can activate tissue macrophages to release chemokines (20) and components of the complement activation pathway, releasing chemotactic factors attracting inflammatory cells into the implant site (21).

The initial process depends on the surgical approach, where a quick resolution of the inflammatory phase is ideal for encouraging the subsequent reparative phase of wound healing (18). This inflammatory local environment drives macrophages to acquire a pro-inflammatory phenotype (M1), causing acute inflammation (2, 4, 22). The resolution of this process leads to acquiring the anti-inflammatory (M2) phenotype, which enhances wound healing (23). Excessive levels of inflammation provoked by the surgery can lead to tissue/biomaterial damage, maintenance of the M1 phenotype, excessive scar tissue, or fibrous encapsulation by excessive fibroblast proliferation with collagen deposition (19, 23).

The latter tissue reaction, driven by several non-immune, immune, and resident cells in the acute postoperative inflammation phase, involves total joint arthroplasty’s encapsulation by a fibrous capsule membrane consisting of a dense collagen network associated with fibroblasts (17, 24). The wound healing process is coordinated in a spatial and kinetic manner by the preoperative planning to achieve the optimal implant position, avoiding chronic maladaptation by innate immune cells (25), which can resolve most cases of postoperative inflammation if the prosthesis is correctly implanted and infection does not develop (4). Templating the shape, size, and correct position of the implant achieves the best match for the patient and could decrease the wear of the total joint arthroplasty components (25). Thus, the correct orientation of the cross-linked polyethylene liner acetabular cup decreases the risk of dislocation, edge loading, and wearing (26).

The present manuscript aims to highlight the contribution of immune cells in the aseptic loosening of the implant by periprosthetic osteolysis. Illustrating the inflammatory cells is an exciting opportunity to decipher the biological mechanisms associated with prosthesis loosening and wear debris particles, identifying new therapeutic targets for developing preventive therapies.

## 2 Early rejection of total joint arthroplasty

When complete osseous integration is achieved, it is observed at magnetic resonance imaging (MRI) as direct contact between the implant/cement and the surrounding trabecular bone (10). However, the most common early failure mechanism in total knee arthroplasty within 2 to 5 years is infection (27), followed by aseptic loosening (28). Periprosthetic joint infection is a rare event occurring in <1%–2% of primary arthroplasties (29). The early and delayed infections, depending on the microbial virulence, are usually acquired through intra-operative inoculation of microorganisms. In contrast, late infections are predominantly acquired by hematogenous seeding at least 3 months post-surgery and during the entire lifetime of the implant (30, 31). This process is enhanced with the presence of a foreign body, such as the implant, which enhances the minimal infecting dose of *Staphylococcus aureus* due to a locally acquired immune defect. Thus, granulocytes show decreased phagocytic activity called “frustrated phagocytosis”. In addition, activation of granulocytes on foreign surfaces leads to the release of human neutrophil peptides defensins that deactivate the granulocytes (30, 32).

However, the reports of early aseptic loosening of total knee arthroplasty show a fixation failure at the cement–implant interface with an intact cement–bone interface with no tibial implant subsidence, probably due to cement–implant debonding and techniques of implantation (33, 34). In some case reports, metal, polymethyl methacrylate, and polyethylene debris were found at revision time (34, 35). Thus, with the development of pulsed lavage techniques and pressurization, the cement penetration into the bone appears to have been improved (36). However, cases of tibial aseptic loosening at the implant–cement interface have shown typical radiographic patterns with debonding at the cement–implant interface, where the bone cement seemed non-adherent to the tibial tray during revision surgery (37).

## 3 Late rejection of total joint arthroplasty

Considering that early implant loss is a rare adverse event, at least 100,000 patients for each million total hip arthroplasty may undergo prosthesis replacement surgery within the first 15 years of service (38). Importantly, it has been observed that an increasing number of younger patients receiving total joint arthroplasty show higher failure rates as they are more active (39). The most common cause of late revision of a total joint arthroplasty is the aseptic loosening of the implant (28, 40–42), accompanied by periprosthetic osteolysis (38). The mechanisms underlying the two last processes are strongly evidenced by chronic low-grade inflammation caused by the contact between wear debris and immune cells (7–11, 13). When wear debris comes into contact with innate immunity receptors of immune cells, it triggers an acute inflammatory response by activated macrophages, fibroblasts, and multinucleated giant cells, increasing osteoclast activity (43–46). Due to the large areas of chronic inflammation of metal hypersensitivities, fibrosis, necrosis, pseudotumor formation, degradation of bone, and aseptic lymphocyte-dominated vasculitis-associated lesions caused by toxic byproducts on cells, newer biomaterials are succeeding, such as highly cross-linked polyethylene and ceramic materials, leaving behind the metal-on-metal surfaces (4, 39, 47).

The intensity and the characteristics of the inflammatory response depend on the material and size of the particles released by the prosthesis materials (48). These implants wear debris from polyethylene, metallic alloys, ceramics, and polymethyl methacrylate cement (49) and are generated by abrasion, adhesion, tribocorrosion, mechanically assisted crevice/fretting corrosion, and pitting corrosion (50–52). The differences between the shape, size, and chemical composition of the implant wear debris observed in the histological examination of the capsular neo-synovial membrane and the “synovial-like” membrane depend on the intensity of the mechanical stress involved (2, 15, 24, 49). In particular, their size and irregular surface lead to an increase in macrophage activation *in vitro* (53). A unified range of measures of polyethylene and metallic particle length only has been proposed, which includes sizes between nanoparticles (1 to 100 nm), submicron particles (>100 nm to <1 μm), microparticles (1 μm to 10 μm), macroparticles (>10 μm to 100 μm), and supra-macroparticles (>100μm) in an attempt to predict their functional biological activity (49). However, the metallic and polyethylene wear particles are produced mainly in nanometric and submicron sizes, which makes their analysis difficult under light microscopy. The process of particle identification then remains challenging (49).

## 4 Mechano-biology of periprosthetic osteolysis

Although the research on periprosthetic osteolysis has been focused on wear debris disease and bone degradation by inflammatory responses, the mechanical factors of the implant designs have been associated with bone loss around the total joint arthroplasty (54, 55).

The surgical procedure relies on stabilizing the implant depending on the design, bone cement, and material (48, 56). Thus, cementless prostheses rely on osseointegration between the bone and the implant. Compared to cement components, this could cause early migration at 1 year, even when stabilization reaches 6 months post-surgery (56). Likewise, implant micromotion might increase wear at different rates according to the configuration and materials of the implant. This micromotion of the implant is essential in prosthesis failure due to their non-homogeneous force distribution over the stem, resulting in abnormally high shear and compressive stresses within the interface (48, 56, 57). However, the mechanisms by which the mechanical forces contribute to periprosthetic osteolysis have yet to be understood entirely.

**Figure 1:**
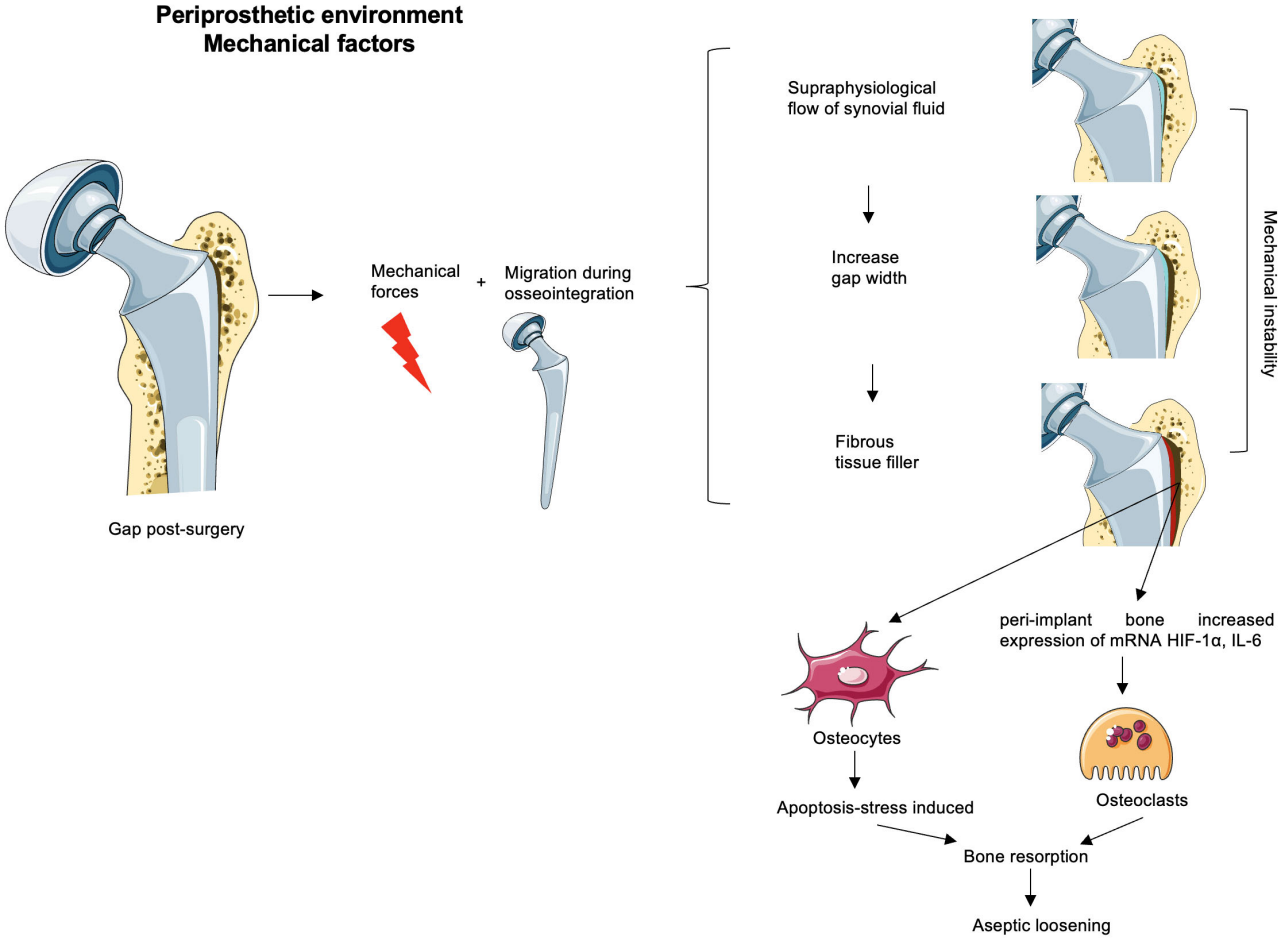
Mechanical factors possibly involved in the aseptic loosening. The remaining gaps after surgery loaded with the mechanical forces and implant migration before osseointegration time increase micromotion. If the initial phase appears unnoticed, the progressive release of wear debris is associated with synovitis corresponding to an inflammatory environment created in the neo-synovium, which leads to an increase of synovial fluid. This increased pressure could drain through the bursa or periprosthetic tissues or remain within the capsule and generate supraphysiological pressures associated with osteolysis and/or flow of synovial fluid in the gaps. This stress maintained over time might increase the width of the gap. Thus, the increase in the width of the gaps enhances the mechanical instability that together with the inflammatory microenvironment (due to the debris particulate presence) would generate a favorable environment for bone resorption and consequently an aseptic loss of the implant. Some authors believe that the width gap is filled with fibrous tissue without any clinical evidence of their hypotheses. The peri-implant bone to the gap subjected to this mechanical instability and/or to the supraphysiological pressure of the synovial fluid increases osteoclastic activators such as HIF-1α or IL-6, and osteocyte apoptosis enhances osteoclast precursor differentiation.

The contribution of mechanical forces to periprosthetic osteolysis has been suggested by *in vivo* and *in vivo* animal models through pressure-induced bone resorption, inflammatory processes, and osteoclastogenesis modulation (58–61). Interestingly, some researchers have questioned the role of wear particles in the pathogenesis of periprosthetic osteolysis, being secondary to the mechanical instability of poorly fixed implants. The micromovements may increase fluid pressure at the bone–implant interface (62, 63) based on specific animal models (64, 65), and they could be initiated during or shortly after surgery due to insufficient initial fixation (63). Animal models and computational fluid dynamic simulation studies support that progressive implant fixation loss is driven by increased fluid pressure into the bone–implant interface, causing osteolysis development (**Figure 1**) (61, 66, 67) (68) (57). In human postmortem studies, cemented knee arthroplasty retrievals show interlock loss between the cement and bone under the tibial tray, being associated with increased micromotion between the implant and bone through fluid-induced trabecular lysis (**Figure 1**) (69, 70). The extent of pressure increase from rest was correlated with capsular distension, which can indirectly indicate the presence of synovitis in MRI (10, 68).

These suggestions are contradictory due to the vast existing literature that validates the acute/chronic inflammation upon the contact between macrophages and implant debris (71–76) commonly presented as a late failure in hip arthroplasty (28, 40–42). Undeniably, a clinically relevant association might exist between the early migration of femoral stems and late revision for aseptic loosening (77). However, the mechanism of how this fluid would enter the bone–implant interface is still unknown, and these preliminary results have not been tested in humans.

The development of a “synovial-like” membrane along the bone–implant interface has been described (70), which may limit osseointegration (78). The synovial-like membrane is assumed at MRI by a smooth intermediate- to high-signal-intensity layer interposed between the host bone and the implant/cement (10). Thus, bone resorption may be identified by the thickness of the hyperintense layer in which a 1–2-mm thickness represents fibrous membrane formation, and more than a 2-mm thickness and irregularity indicate bone resorption (10). The mechanical stress may be responsible for the membrane formation, promoting synoviocyte migration into the bone–implant and bone–cement interfaces (79). However, the effect of fibrous membrane formation on implant fixation is uncertain; it may or may not progress to component loosening and may warrant closer imaging surveillance (10). A study suggests that it might transmit pressurized fluid flow with or without the presence of wear debris particles to the bone–implant interface, leading to osteoclastogenesis by the expression of pro-inflammatory genes in peri-implant bone loss (61). Despite the above, mechanical loosening may be inferred by using MRI if laboratory tests for infection are negative and the findings at MRI examination are negative for wear-induced synovitis, which often incites bulky osteolysis (10).

Despite the evidence presented, these studies still need to be more credible due to the need for previous standardization of the tissue samples regarding implant materials, cement, and techniques. It is known that bone is a dynamic tissue responsive to mechanical stimuli, so how accurate are these supraphysiological fluid pressure resorptions on *in vivo* models within the human bone–implant interface, for which there is no clinical evidence of fluid infiltration? (61, 66, 67) It is essential to highlight mechanical events in the pathophysiology of aseptic loosening and to believe that these two factors work in concert in a multifactorial disease (80). The combined effect of wear and increased pressure may contribute to the osteolytic process with a wide variability in the contribution of biological and mechanical factors in aseptic loosening in each patient (33, 34, 37, 80).

Consistently, due to the need for more human evidence, there is no guideline for the ideal frequency and number of postoperative control radiography (81). However, these implants must have long-term surveillance due to the clinical undetectability of progressive wear that can lead to substantial bone loss, resulting in prosthetic interfacial micromovements and loosening or pathological fracture (82). Follow-up of asymptomatic total knee arthroplasty patients by annual radiographs is recommended to identify subtle interval changes or postoperative complications as Aseptic Loosening (AL) (81–83). In contrast, postoperative radiographs may be unnecessary because they do not change clinical management, only indiscriminately irradiating the patient and increasing healthcare costs (84–86). Importantly, MRI with metal artifact reduction sequence (MARS) has not been validated for use in the detection of aseptic loosening (81) but appears to be a reliable method of distinguishing between aseptic complications and infections (87), being able to visualize polyethylene wear-induced periprosthetic synovitis (10, 11).

Adjustments to the composition of the implants, such as cross-linking polyethylene and vitamin E enrichment to increase oxidation resistance or replacement with novel polymers, aim to improve patient outcomes by reducing the production of wear debris particles. Nevertheless, no device on the market is free of wear debris, and no Food and Drug Administration (FDA)-approved non-surgical pharmacological intervention can arrest particle-associated periprosthetic osteolysis. The challenges associated with biological responses to wear debris are ongoing.

## 5 Immunological pathogenesis of aseptic loosening and periprosthetic osteolysis

In later stages of non-solved inflammation over the years in total joint arthroplasty, wear debris particles activate the innate immune response characterized by the foreign body chronic inflammatory response (88). As a continuation, we will discuss each cell population that participates in this response.

### 5.1 Macrophages and periprosthetic osteolysis

The ancient dogma on the origin of macrophages, when describing the mononuclear phagocyte system (MPS) theory (89), concluded that monocytes develop as precursors in the adult bone marrow and then enter into circulation to constantly replenish macrophages in the tissues. However, the current paradigm with newly published evidence of local proliferation (90) and the self-renewal capacity of macrophages of different tissues (91, 92) holds that most macrophages are tissue-resident macrophages developed during embryogenesis and self-renew in most tissues without inflammatory stimuli or severe depletion (93–95). This mechanism of how tissue-resident macrophages are tissue residents also might apply to the synovium.

In the periprosthetic environment, macrophages are monocyte-derived macrophages recruited from the bloodstream and bone marrow for being involved in immune surveillance such as type A synovial lining cells, osteoclasts, and connective tissue histiocytes (4, 22, 23, 76, 96–98). After a total joint arthroplasty, particles are continuously generated and dispersed in the periprosthetic tissues due to ongoing wear between implant components. Consequently, accumulating particle debris activates macrophages CD68^+^ (45, 88, 98–100). The activation of macrophages at the implant–tissue interface can occur by “failed phagocytosis” of indigestible wear particles to become foreign body giant cells (4, 24, 76, 98, 101) or by cell contact through toll-like receptors (TLRs) 2 and 4, CD11b, and CD14 (4, 22, 38, 102). In aseptic loosening, the immune reaction was usually observed in synovial membrane-line interface tissues by co-localizing polymeric particles and macrophages (97, 98, 103).

The macrophage activation results in the release of inflammatory cytokines, chemokines, reactive oxygen species (ROS), tumor necrosis factor-α (TNF-α), interleukin (IL)-1β, IL-6, IL-17, and interferon-γ (IFN-γ), which are potent contributors to bone resorption (2, 4, 22, 102, 104–108). TLRs and nucleotide-binding oligomerization domain (NOD)-like receptors (NLRs) can recognize exogenous pathogen-associated molecular patterns (PAMPs) and endogenous molecules created during inflammation and tissue remodeling. These events increase local host response and maintain the chronic periprosthetic inflammation (4). However, the exact mechanisms of how wear particle-induced macrophage activation translates signals into a biological response remain unclear. It has been reported that Cobalt ions (Co) can activate TLR4-positive cells such as macrophages and dendritic cells (109). Recently, studies suggested that metallic particles can activate the inflammasome NLRP3, inducing the secretion of IL-1β *in vitro* human macrophages with titanium (Ti), chromium (Cr), and molybdenum (Mo) with the help of an additional priming signal that could be TNF-α in replacement of lipopolysaccharide (LPS) in the aseptic environment (110, 111).

**Figure 2:**
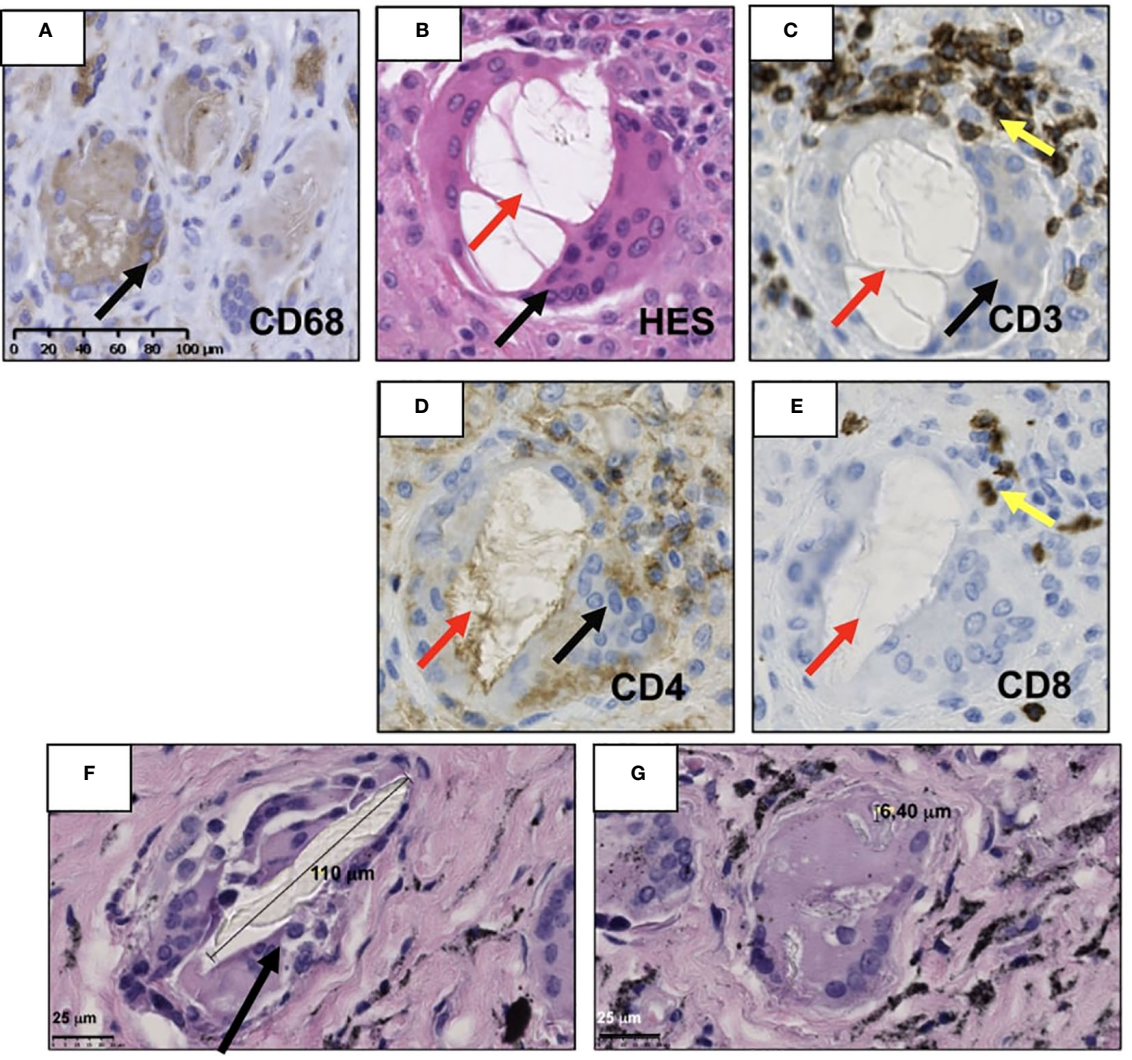
Illustration of the periprosthetic neo-synovium membrane retrieved during the revision of total joint arthroplasty patient with clinic–radiographic and microbiological evidence of aseptic loosening. **(A)** Presence of macrophages CD68^+^ (black arrow) surrounding cement particles. **(B)** Vacuole of orthopedic cement dissolved (red arrow) due to tissue processing lined by multinucleate giant cells and CD68^+^ (black arrow). **(C)** Human T lymphocytes detected by immunohistochemistry using primary anti-human CD3, **(D)** CD4, and **(E)** CD8 antibodies (yellow arrow) (Abcam, Cambridge, MA, USA) were recognized in close contact with multinucleated giant cells (black arrow) but are not specific for this condition. **(F, G)** Hematoxylin–eosin saffron (HES) staining of retrieved neo-synovium of 9.6 survival years of non-metal-on-metal implants. The metallic debris of high Ti content is secondary to a massive bearing failure by impingement loading over time. **(F)** Multinucleated cells (black arrow) phagocytize supra-macroparticles probably produced by delamination and **(G)** microparticles of polyethylene. The histological images of immunochemistry were acquired using the digital slide scanner NanoZoomer 2.0-RS (Hamamatsu, Japan) and then exported as a ×10 field using viewing software NDP.view2 (Hamamatsu, Japan).

In this inflammatory local environment, which drives macrophages to acquire a pro-inflammatory phenotype (M1) (2, 4, 22), polyethyl methacrylate wear particles around the prosthesis have been reported to enhance M1 macrophage expression and lower expression of M2 anti-inflammatory macrophages, increasing the local inflammation (23). Likewise, in cemented prosthesis, macrophages were observed in an attempt to phagocyte orthopedic cement particles (**Figure 2**) and stimulate high-level TNF-α release, enhancing the pro-inflammatory environment (45, 49, 75). In contrast, an *in vitro* study shows that the expression of IL-4 in the presence of polyethyl methacrylate M1-induced macrophage can polarize M2 (23).

The adverse local tissue reaction and adverse reaction to metallic debris in aseptic loosening (112) are used as equivalents for metal-on-metal implants and non-metal-on-metal prostheses with metallic junctions, which by definition can produce only metallic wear debris by corrosion (72).

The adverse local tissue reaction/adverse reaction to metallic debris reactions can show different features. One of them is a *pseudotumor* of periprosthetic soft tissue in metal-on-metal implants characterized by a mass of variable size. This feature is a reactive proliferation of the joint pseudo-capsule and neo-synovial membrane with or without a tissue necrosis/infraction layer and with a variable amount of synovial fluid (72, 113). The early onset of adverse local tissue reaction/adverse reaction to metallic debris pseudotumor is characterized by the presence of macrophage infiltration with metallic particulate debris and requires the presence of perivascular lymphocytic components. The progression to an advanced stage of this type of pseudotumor may show an adverse reaction with soft tissue necrosis (72, 112).

**Figure 3:**
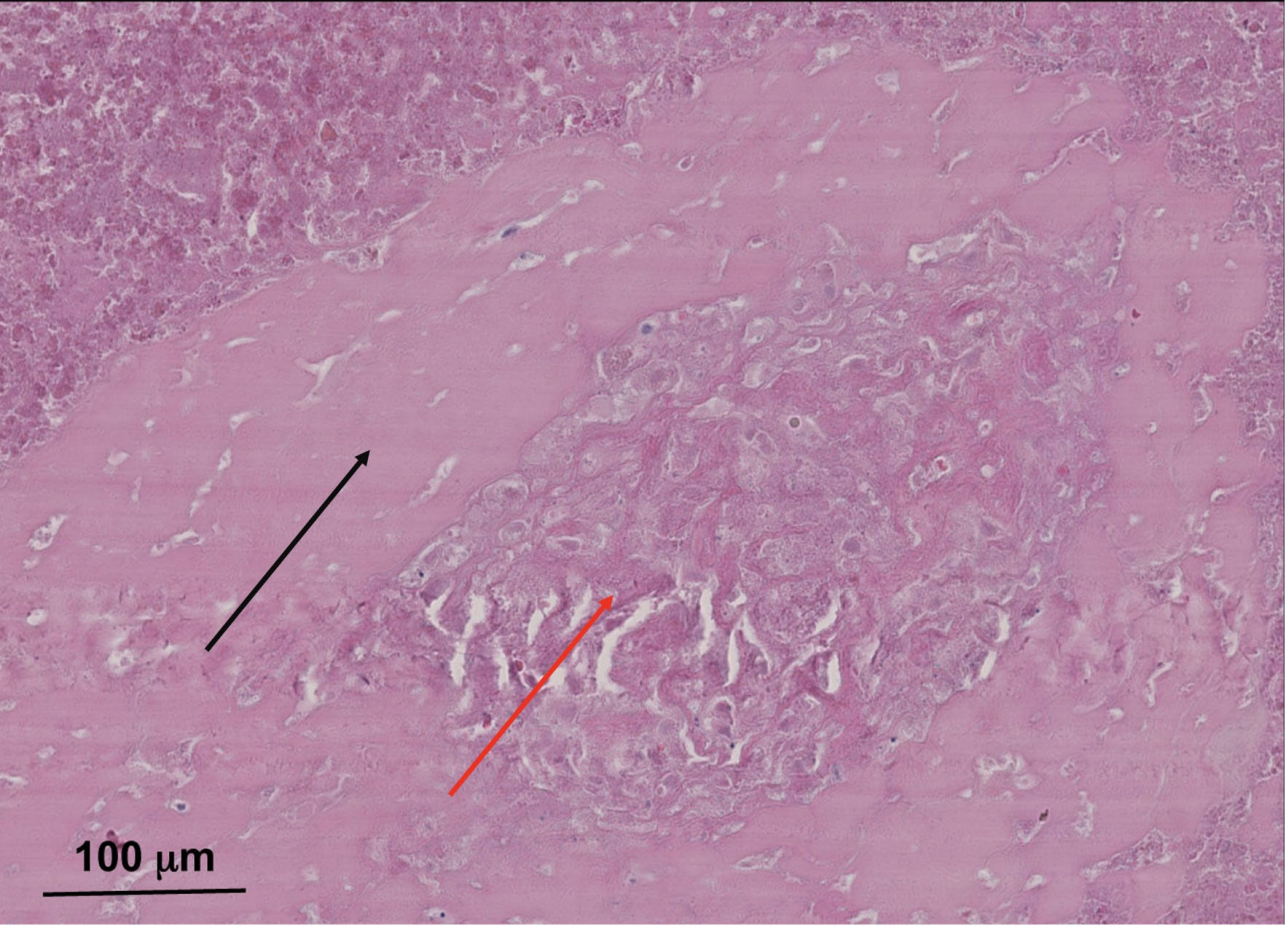
Typical necrotic pseudomembrane associated with pseudotumor in metal-on-metal total hip arthroplasty with clinic–radiographic and microbiological evidence of aseptic loosening. Sample from 83-year-old male patient with eight survival years on metal-on-metal implant. Hematoxylin–eosin saffron (HES) staining images were acquired using the digital slide scanner NanoZoomer 2.0-RS (Hamamatsu, Japan) and then exported as a ×10 field using viewing software NDP.view2 (Hamamatsu, Japan). Soft tissue necrosis with a band of dense connective tissue (red arrow) surrounded by wear debris particles in necrotic foci (black arrow). This patient’s lack of more histological samples did not allow for a diagnosis. However, it appears that there was an advanced stage of adverse local tissue reaction/adverse reaction to metallic debris with soft tissue necrosis. This is not specific to metal-on-metal implants and metallic corrosion particles; however, this necrosis can be present in pseudotumors.

However, the late onset is characterized by a slow reactive proliferation of the neo-synovium capsule with exclusive macrophage infiltrate of the bone marrow and fibrovascular stromal proliferation with a minimal lymphocyte infiltrate. This late onset can lead to significant clinical particle-induced periprosthetic osteolysis (72, 112). However, necrosis can be observed in the case of adverse local tissue reaction/adverse reaction to metallic debris (**Figure 3**), predominantly of macrophages in the periprosthetic neo-synovium. Also, it can be seen in polyethylene wear under polarized light (72).

Wear of polyethylene causes histiocyte-mediated synovitis (114), which at MRI is characterized by an expansion of the hip pseudo-capsule by thick synovitis of low intermediate signal intensity (10). In metal implants articulating with polyethylene capsules, the macrophages CD68+ are located beneath the synovial cell lining or fibrin layer, which is adjacent to the surface of the implants, meaning that polyethylene particles can migrate into the surrounding soft tissues (97). In supra-macroparticles (>100 μm) of polyethylene, the multinucleated giant cells (**Figure 2**) are free in the stromal tissue or surrounded by particles recognized as CD68^+^ and TRAP^−^ (49) (**Figure 2**).

Thus, the pro-inflammatory cytokine and chemokine system becomes upregulated, causing local tissue destruction and encouraging the regional migration of other inflammatory cells to the area. These events, together with the release of wear debris, are a continuous process throughout the life span of implants, fueling a persistent pro-inflammatory periprosthetic environment that alternates between acute and anabolic responses. Subsequently, these alternations result in a cellular reaction that has variable composition and degree, depending on the particulate wear debris, host factor, and time of implantation (4, 96). Moreover, periprosthetic stromal and bone tissues contribute to implant loosening by secreting soluble factors (RANKL, IL-1β, and IL-6), promoting the differentiation of myelomonocytic-derived cells into bone-resorbing osteoclasts and their subsequent activation (14, 115). These secretions and consequent osteoclast activation can cause periprosthetic osteolysis, ultimately leading to AL (15, 105). Although pro-inflammatory cytokines usually allow mesenchymal stem cells (MSCs) and vascular progenitors to initiate the reparative process, the continued pro-inflammatory environment supersedes these pro-reconstructive events for the lack of polarization of the macrophages, as occurs in wound healing (4, 116, 117).

### 5.2 Foreign body reaction: giant cells

**Figure 4:**
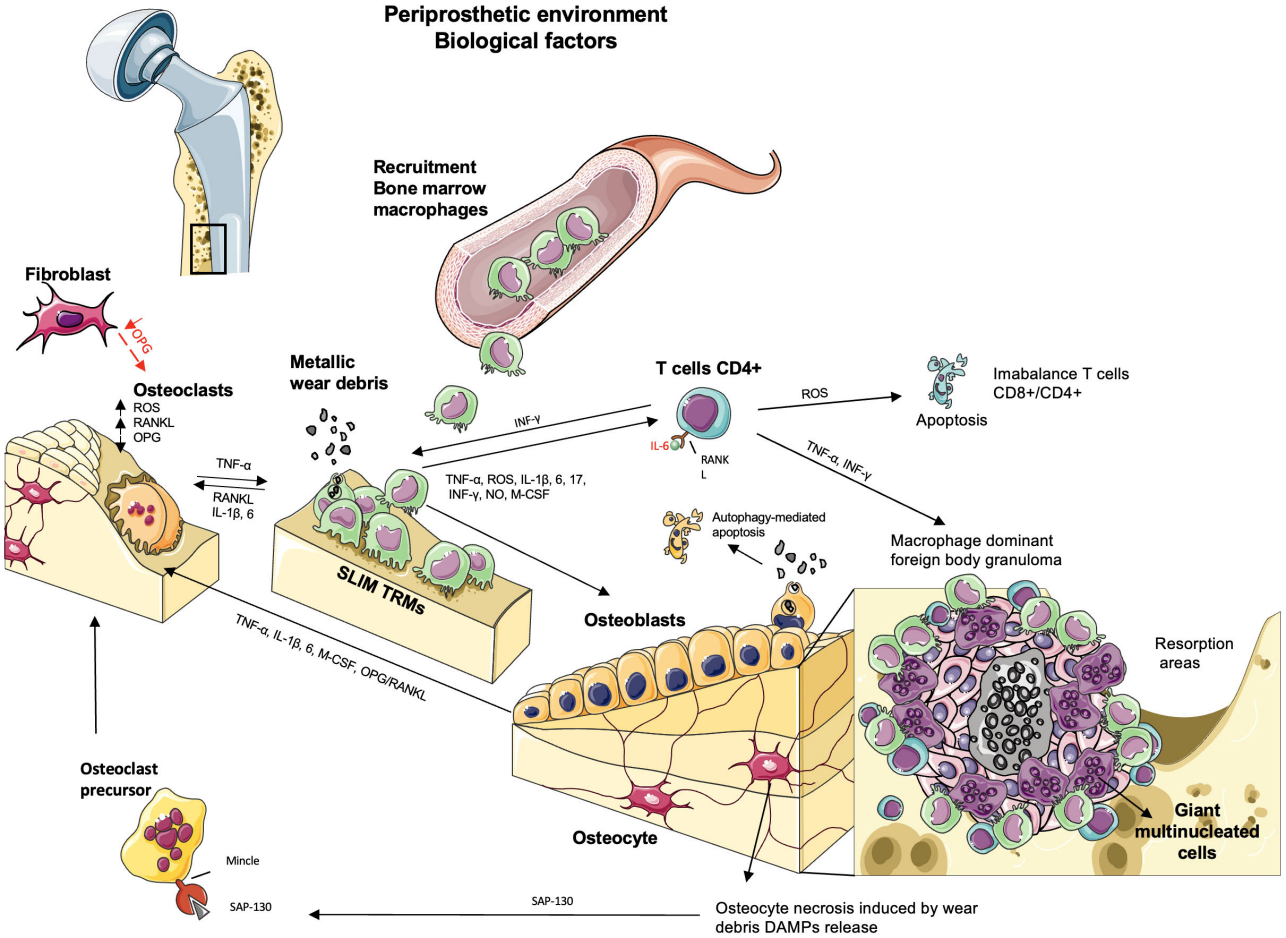
The biological factors involved in aseptic loosening by particle-induced periprosthetic osteolysis. The wear debris produced by abrasion or corrosion in the bone–implant interface activates resident macrophages (“synovial-like membrane”), fibroblasts, and multinucleated giant cells in the soft tissues, releasing TNF-α, IL-1β, 6, 17, IFN-γ, and M-CSF. The persistence of this inflammatory state activates the acquired immune response where the recruitment of bone marrow macrophages and tissue-resident macrophage (TRM) synovial-like membrane macrophages produces reactive oxygen species (ROS) and NO, contributing to osteoclast differentiation and inducing apoptosis in T CD4^+^ lymphocytes. The presence of lymphocytes is due to metallic debris/ions in the periprosthetic soft tissue or recruited by the macrophages in a chronic inflammation that triggers adaptive immunity. The presence of the granulomatous reaction is a specific response in presence of metallic debris observed in a small number of patients where the pro-inflammatory mediators and proteases cause a macrophage-dominant foreign body granulomatous reaction. Ischemia and necrosis are observed in the deeper layers of granulomas, whereas lymphocyte infiltration is occasionally observed with numerous giant cells. The central necrosis may lead to release of damage-associated molecular patterns (DAMPs) to the inflammatory environment and trigger osteoclast differentiation and bone loss in the periprosthetic bone tissues adjacent to foreign body granulomas. Osteoclasts are activated by RANKL. OPG acts as an inhibitor of RANK–RANKL signaling, which is expressed by fibroblasts. However, in the periprosthetic environment, there is a downregulation of OPG, and the local expression of RANKL and ROS upregulates the differentiation of osteoclasts and bone resorption. Osteoblasts regulate osteoclasts by secreting RANKL and OPG. Wear debris activates the secretion of TNF-α, IL-1β, IL-6, and M-CSF, increasing osteoclast activity and leading to bone loss, but their internalization leads to changes in osteoblast functions or autophagy, which promotes osteolysis. When osteocytes undergo necrosis, they release DAMPs, triggering osteoclast differentiation and bone loss.

Over time, chronic inflammation in the periprosthetic tissues activates the innate immune system through the foreign body granuloma response (38, 47, 75, 118). This reaction is driven mainly by the foreign body multinucleated giant cells being the macrophage–macrophage fusion in response to larger particles or when there is an insufficient primary mechanism of material degradation with smaller particles. They are associated with fibrous encapsulation and physical walling around the implant, preventing the appropriate molecular transport and vascularization and maintaining an internal wound until removed (119). Before the macrophage fusion, through exogenous stimuli, the mechanical (120) and biomolecule signaling (121–123) lead to an efficient fusion competency of macrophages by the expression of fusogens enhancing the cell–cell attraction (121, 124). Thus, granulomatous inflammation is an adverse host tissue response characterized by angiogenesis and connective tissue proliferation with the presence of ischemia and necrosis in the deepest layers by the excessive death of immune cells and the difficulty of their removal by phagocytic cells (4). Over time, the insufficient removal of these apoptotic cells results in secondary necrosis and the consequent formation of necrotic tissue (**Figure 4**) (4, 125).

In the macrophage-dominant foreign body granuloma with numerous giant cells, lymphocyte infiltration is occasionally observed (4, 47). Instead, lymphocyte-dominant tissue reaction is observed predominantly in metal-on-metal total joint arthroplasties, particularly concerning hypersensitivity (38, 73). Adjacent to the foreign body granuloma, osteoclastic bone resorption is observed in the periprosthetic bone tissues through the imbalance in favor of osteoclast number/activity, thanks to maintaining this chronic inflammatory environment (38). Also, the high concentrations of auto-activated cathepsin K, a matrix-degrading enzyme found in peri-implant tissues and fluids, enhance bone loss, resulting in prosthetic loosening (126, 127).

### 5.3 Role of T lymphocytes in particle-induced periprosthetic osteolysis

Despite the critical role of the lymphocyte cells in sustaining the adverse reactions in metal-on-metal implants (4, 72, 128–130), in several pathophysiological processes, the balance of the different T-cell subsets influences the healing outcome. One subset is regulatory T cells, a subset of CD4^+^ T lymphocytes that inhibit osteoclast differentiation from peripheral blood mononuclear cells by producing IL-4 cytokine (131). An impaired function or lack of CD4^+^ T-cells leads to diminished wound repair. CD4^+^ Th1 cells, through the production of IFN-γ, promote the activation of macrophages, create a “positive loop” in inflamed synovia, and suppress RANKL expression in T cells in orthodontic animal models (132). Also, metallic particles in metal-on-metal implants act as antigens by T cells in a type IV delayed hypersensitivity reaction, activating a local or systemic inflammatory reaction and releasing osteoclastogenic cytokines (133, 134). However, in the advanced stages of aseptic loosening, the evidence is controversial due to the low number of lymphocyte T in the samples (135).

Evidence shows an increased T-cell number in osteoarthritis with more CD4^+^ T cells than CD8^+^ T lymphocytes and changes at inverse in patients with total joint arthroplasties for particle-induced periprosthetic osteolysis. The apoptotic reaction of CD4^+^ T lymphocytes in the capsules and interface membranes is induced by the increased expression of iNOS and ROS by macrophages (115, 118, 136), creating an imbalance in the CD4^+^/CD8^+^ ratio, which suggests a correlation with the stage of osteolysis in aseptic loosening. The increase in CD8^+^ cells affects mechanical strength, and the apoptotic reactions in CD4^+^ T cells are harmful by activating osteoclasts (118).

A variable presence of lymphocyte infiltrate (49, 72) in the adverse local tissue reaction/adverse reaction to metallic debris in aseptic loosening and a lymphocytic infiltrate does not mean direct prosthetic joint infection (71). Indeed, the apoptosis of lymphocytes may explain the increase of the osteoclast activity in the periprosthetic bone due to the lack of releasing IL-4, a protector of osteoclast activity (118). The central stroma of pseudomembranes comprises highly vascularized and cell-infiltrated fibrous tissue. Lymphoid cells were recognized in perivascular sites throughout the pseudomembranes (47, 72, 137–139). Moreover, T lymphocytes (CD3^+^, CD4^+^, and CD8^+^) are in close contact with the multinucleated monocyte/macrophage cells (**Figure 2**) surrounding large polyethylene particles. CD3^+^, CD4^+^, CD8^+^ T, and CD20^+^ B lymphocytes were identified, forming a perivascular syncytium with a majority of CD3^+^ foci compared to CD20^+^ areas (47, 112).

In particular cases of adverse local tissue reaction/adverse reaction to metallic debris (112), polyethylene debris in total knee arthroplasty implant with regional lymph node involvement (140), and massive metallic wear debris with lung involvement (141) patients, a distinctive type of foreign body reaction is observed (112), characterized by a granulomatous reaction restricted to sarcoid-like epithelioid cell granuloma with giant cells containing or surrounding particulate material with or without the presence of an incomplete/complete lymphocytic or lymphoplasmacytic cuffing (72). It is essential to distinguish between infections and rheumatoid nodules due to the possible misdiagnosis of lymphoplasmacytic infiltrate and granulomas in non-metal-on-metal total hip arthroplasty (71).

Recently, preliminary results of long non-coding RNAs (lncRNAs), recognized as crucial regulatory molecules with diverse roles in gene expression, epigenetic modification, and protein activity, were revealed to be involved in osteolysis. Even two shared lncRNA–mRNA interaction pairs in osteoarthritis and osteolysis (AC111000.4 and AC016831.6) may function in the immune process of osteoarthritis and osteolysis by regulating lymphocyte CD8A and CD8B, respectively. Two osteolysis-specific interaction pairs (AC090607.4-FOXO3 and TAL1-ABALON) may be essential in osteoclastogenesis. LncRNA TSIX was involved in particle-induced osteolysis by regulating miR-30a-5p to promote osteoblast apoptosis. LncRNA DANCR inhibits osteoblast differentiation in osteolysis after total hip arthroplasty through holding FOXO1, and lncRNA KCNQ1OT1 may improve particle-induced osteolysis by inhibiting miR-21a-5p, inducing macrophage polarization (142).

### 5.4 Functional contribution of B lymphocytes in particle-induced periprosthetic osteolysis

Lymphoid CD20^+^ B cells have been seen in perivascular sites through the pseudomembranes, forming perivascular syncytium in a minor quantity of CD20^+^ compared to CD3^+^ cells, suggesting activation of the acquired immune response (24, 47, 101, 112, 143). However, B lymphocytes were predominantly seen in cases with periprosthetic joint infection (144).

Several vascular changes have been described in the local adverse tissue reaction (72, 101, 145), in which the onion skinning pattern, the occlusion of the lumen, and capillary/venule wall thickening can be observed in high endothelial venules but non-specific of adverse local tissue reaction/adverse reaction to metallic debris (72, 101). The relationship between laden macrophages, wear debris, and the vascular changes with the subsequent lymphocyte migration and trafficking should be studied in depth to better understand the lymphocytic migration and the role played by large-vessel involvement (72, 101).

### 5.5 CD117^+^ mast cells in response to wear particles

Mast cells (MCs) are characterized by releasing IL-1, IL-6, and TNF-α implicated in bone resorption. MCs had been concentrated at the bone interface and appeared to be degranulated, indicating high activity of MCs and release of tryptase at the regions of bone destruction (146). A mixed macrophage and lymphocytic infiltrate pattern has been described as generally associated with hypersensitivity reactions, which advocated the inflammatory response to wear particles (112, 128). This pattern is characterized by the presence of a large number of CD117^+^ mast cells in association with macrophages or perivascular cells (112, 147). Furthermore, the periprosthetic tissue of 31 patients with total joint arthroplasty was examined, the increased density of mast and dendritic cells was associated with polypously formed pseudosynovium, and cement fixation prostheses may be due to the reaction induced by cement particles (148). However, the interaction between the mast cells and the wear particles is not well elucidated.

Therefore, there could be an indirect potential involvement of CD117^+^ mast cells in periprosthetic osteolysis by releasing inflammatory factors that contribute to bone resorption under wear debris.

### 5.6 Osteoclasts: the pivotal cells in charge of bone resorption

There is strong evidence that osteoclasts play a significant role in particle-induced periprosthetic osteolysis. Regardless of the biological mechanisms leading to osteolysis, osteoclasts are the central bone-resorbing cells (149), and the intensity depends on the number, activity, and survival of osteoclasts (38, 102).

Osteoclasts are multinucleated cells from mononuclear/macrophage progenitors. After macrophage recruitment began with the release of M-CSF, the presence of RANKL promotes its differentiation into osteoclasts (95, 102, 115). The RANK–RANKL–OPG pathway explains these cells’ formation, activation, and survival (102, 115). OPG acts as an inhibitor of RANK–RANKL signaling and, in periprosthetic tissues, is expressed by vascular endothelial cells and fibroblasts, inhibiting osteoclastic activation (150). In aseptic loosening, there is an increase in the RANKL/OPG ratio due to an increase of RANKL and a downregulation of OPG. Although the cell source of RANKL is not fully verified, their expression has been localized in fibroblast-marked cells (151). Thus, the local expression of RANKL upregulates the differentiation of osteoclasts and bone resorption (151, 152).

Bone homeostasis is the result of the balance between bone formation and resorption. Following the chronic inflammation caused by wear debris could be an over-activation of osteoclasts synthesizing cysteine proteinases (e.g., cathepsin K) and metalloproteinases (MMPs) that cause bone resorption in cases with clinical osteolysis (151). The imbalance between bone metalloproteinases and their inhibitors is also a result of chronic inflammation (38, 153). Specific matrix metalloproteinases, such as MMPs 1, 2, 9, and 13, can be overexpressed at the bone–implant interface, contributing to the growth of particle-induced periprosthetic osteolysis in this way (38, 88). In addition, elevated mitochondrial ROS levels are essential for hypoxic osteoclast differentiation by the release of Ca^2+^ and induction of a RANKL-independent activation of NFATc in osteoclasts, contributing to particle-induced periprosthetic osteolysis and aseptic loosening (38, 64, 115) (**Figure 4**). Surprisingly, osteoclasts can also secrete extracellular exosomes and microvesicles to regulate osteoblasts. However, the role of these vesicles in the context of aseptic loosening is still unknown (2).

## 6 Osteoblasts: a contributor to bone resorption

In bone homeostasis, osteoblasts deposit bone through the ossification process, regulated by the degradation of osteoclasts for balance maintenance (2, 104, 115). Osteoblasts also participate directly in bone resorption by secretions of pre-osteolytic mediators and proteinases and indirectly by expressing specific chemokines or changing cell viability (2, 115). They come from mesenchymal stem cells, and their maturation from osteoblast progenitors is characterized by an increase in the expression of osterix (OSX), osteocalcin (BGP), bone sialoprotein I/II (BSP), and collagen type I (2, 115). Differentiation of osteoblasts is driven by runt-related transcription factor 2 (runx 2), WNT, and bone morphogenetic protein (BMP) signaling pathways. It has several possible outcomes: osteocyte differentiation, apoptosis, or inactivation into quiescent bone-lining cells. Osteoblasts also regulate osteoclasts by secreting RANKL and OPG (2, 115). Recent *in vitro* studies show that wear debris can also inhibit osteoblast function and disfavor new bone formation, playing a synergic role by coordinating with macrophages and osteoclasts during osteolysis (2, 14).

Experimental studies *in vitro* in need of confirmation in human tissue have demonstrated that osteoblasts internalize wear debris within the cytoplasm through several pathways, such as contact, endocytosis, and micropinocytosis (2, 13, 14). Once they are engulfed in particles, osteoblasts exhibit structural changes in their organelles, with an impact on osteoblastic functions such as proliferation, adhesion, and migration, depending on the composition of the particles, size, time, and doses (2, 14).

Autophagy is a catabolic and evolutionarily conserved process in eukaryotes, which plays a role in the survival response to wear debris particles. However, some studies suggest autophagy modulates osteoblastic function; cell death occurs when the protective effect is limited (154, 155). In an animal model, it has been seen that autophagy-mediated osteoblast apoptosis promotes osteolysis *in vitro* and *in vivo* (2, 154, 156). Osteoblast exposure to wear particles impairs mineralization by reducing the gene expression of ALP, runx 2, osterix, and late osteogenic markers such as osteocalcin (157) and impairs the capacity to synthesize type I collagen, the particle-induced inhibition of osteogenic differentiation by WNT/β-catenin and BMP/Smad signaling pathways, and the imbalance between osteoblastic MMPs and tissue inhibitors of metalloproteinases (TIMPs) (158). This imbalance could result in limited osseointegration and consequently the loosening of the implant (158).

However, osteoblasts contribute to peri-implant inflammation. Wear debris in a time- and dose-dependent manner activates osteoblasts to secrete inflammatory mediators such as TNFα, IL-1β, IL-6, and M-CSF, increasing osteoclast activity and leading to bone loss. Their importance in periprosthetic tissue relies on TNF-α control of the release of IL-1β and IL-6; both TNFα and IL-1β alter collagen matrix formation by osteoblasts (2, 14, 104). Likewise, osteoblasts play a role in the local and systemic recruitment of inflammatory cells through the production of chemokines, such as monocyte chemoattractant protein-1 (MCP-1, also known as CCL2) and IL-8 (also known as CXCL8) in responding to wear debris particles (2). Macrophages can produce osteoblast activity factors such as BMP-2 and TGF-β (2). During wear debris-induced periprosthetic osteolysis, the macrophages are recruited to the local site (13, 32, 44). In contrast, bone tissue-resident macrophages (OsteoMacs), essential in directing osteoblast function/mineralization, have not been studied during AL (2). Osteoblasts also interact with osteoclasts by secreting RANKL and OPG to maintain the balance and particle-induced mature osteoblastic secreting inflammatory mediators, as mentioned above (2, 156, 159).

*In vitro* studies have demonstrated significantly elevated RANKL gene expression and OPG gene suppression, producing an imbalance in the RANKL/OPG ratio, which leads to particle-induced periprosthetic osteolysis through a RANKL-dependent pathway in particle-induced osteoblasts (2, 13, 159, 160). Also, the increased expression of genes promoting osteoclast formation and activity with RANKL, M-CSF, and IL-8 and the decreased expression of OPG mRNA exacerbated osteoclastic bone resorption (2, 159). Likewise, mature osteoblasts exposed to wear debris showed apoptosis and increased mRNA expression of inflammatory cytokines, E11, DMP1, and SOST *in vitro* (2). Although wear particle internalization is vital for a cellular reaction, more research is required on these cells and their role in AL (**Figure 4**).

## 7 Osteocytes: modifiers of the microenvironment and bone resorption

Osteocytes are the most numerous cells in bone tissue originating by their differentiation from osteoblasts embedded in lacuna within the mineralized bone matrix (2, 64, 115). They are described as both sclerostin-secreting cells that inhibit osteoblast activity when osteon reaches a limiting size and RANKL-secreting cells, helping osteoclastogenesis (64, 115). Like osteoblasts, osteocytes have been shown to respond to wear debris and contribute to peri-lacunar remodeling, a particular type of bone loss, by the expression of cathepsin K and tartrate-resistant acid phosphatase (TRAP) *in vitro*. Although there are reports of a significant increase in osteocyte lacunar size induced by wear debris, it is suggested that osteocytic bone resorption may be specific to female people (2).

Also, osteocytes undergo autophagy when there is excessive damage, such as disruption of the canalicular flow and decreasing oxygen and nutrients; osteocytes undergo cell death secondary to necrosis in their lacunae in *in vitro* studies (2, 64), where undergoing necrosis releases damage-associated molecular patterns (DAMPs) into the environment and triggers osteoclast differentiation and bone loss (64). DAMPs are recognized through pattern recognition receptors (PRRs), mainly expressed in the myelocytic cell lineage, especially by PRR macrophage inducible C-type lectin (Mincle). Mincle on preosteoclasts senses small nuclear ribonucleoprotein SAP-130, released explicitly by osteocytes undergoing necrosis, and induces osteoclast activation and bone loss. This activation occurs by inducing calcium signaling and oxidative phosphorylation in osteoclasts, which provides a RANKL-independent activation of NFATc1 and increased metabolic activity on osteoclasts, respectively. In summary, necrotic osteocytes enhance osteoclastogenesis and bone loss (64) (**Figure 4**).

## 8 Conclusion

In total hip joint prosthetic devices, some variabilities in aseptic loosening may be explained by inter-individual variability: first, the cause of their correction (i.e., pseudotumor with associated fracture, dislocation of the prosthetic femoral head, pain, or osteoarthritis); second, mechanical stabilization through a cementing agent or by osseointegration; finally, the wear debris particles’ morphological heterogeneity (nature, size, and shape) due to their origin in the prosthetic device.

**Figure 5:**
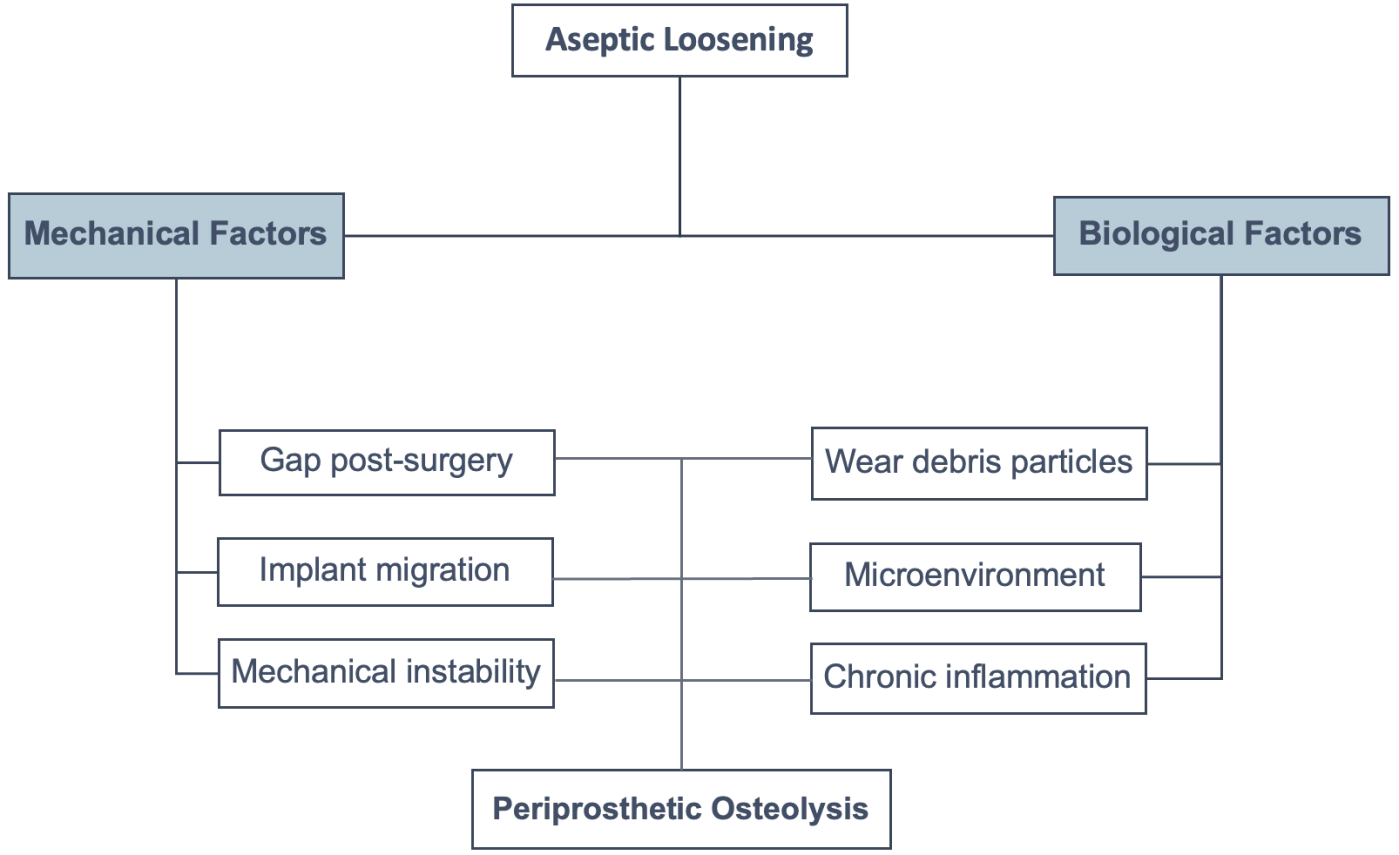
Factors involved in aseptic loosening. There are mechanical and biological factors that enhance particle-induced periprosthetic osteolysis, resulting in aseptic loosening.

Our review illustrates the cause-and-effect relationship between the mechanical and biological factors in particle-induced periprosthetic osteolysis. The new evidence of mechanical instability, the release of wear particles from loosened orthopedic implants, and the triggering of the cellular response in human periprosthetic tissues contribute to particle-induced periprosthetic osteolysis. Although new evidence shows an early role played by mechanical instability in osteolysis activation, which may contribute to aseptic loosening in particle-induced periprosthetic osteolysis, further studies are required. We summarized the new evidence of the biological factors related to these pathological processes. The microenvironment of this chronic inflammation triggered by wear debris directly depends on the prostheses material where macrophages are the first-line cell effectors of innate immunity, present in periprosthetic pseudomembranes engulfing large and small particles of PE, metal, and ceramics (4, 23, 45, 49, 72). Despite the above, recent *in vitro* studies showed the role of osteoblasts in aseptic loosening (2), reminding us that the microenvironment plays a fundamental role in cell fate (**Figure 5**). However, further clinical studies in human patients are mandatory.

Evidence of close contact between CD68^+^ monocytes/macrophages and T cells suggests crosstalk between the lineages (115, 130, 136). However, the activation of lymphocytes occurs in different degrees on particles of debris, and the exaggerated response may increase the presence of necrosis and pseudotumors. The reaction from aseptic loosening and allergic reaction must be differentiated to avoid misdiagnosis (71).

It is worth noting the importance of the microenvironment prior to total joint arthroplasty, which is generally in patients with osteoarthritis. This environment difference with a health joint shows different cellular features than the synovium retrieved from osteoarthritic patients. The chronic inflammation present in osteoarthritis may impact the slow healing of the postoperative inflammation after an arthroplasty. Thus, maintaining the inflammatory environment leads to aseptic loosening over the years.

Overall, this review highlights the role played by immune cells in aseptic loosening. This contribution and the new evidence will guide future research toward a better understanding of the complete process for aseptic loosening. Furthermore, this review will make possible a better understanding of the temporality of aseptic loosening and the role played by particle-induced periprosthetic osteolysis. Immune cells are not only responsible for fueling the dysregulation of bone remodeling but should also be considered as new biomarkers of AL and a source of new therapeutic options.

## Author contributions

IP-T: Writing – original draft. DH: Writing – review & editing. FG: Writing – review & editing. JA: Writing – review & editing. M-FH: Writing – review & editing. LC: Writing – review & editing.

## References

[1] Loeser RF Goldring SR Scanzello CR Goldring MB. Osteoarthritis: A disease of the joint as an organ. Arthritis Rheumatism. (2012) 64(6):1697–707. doi: 10.1002/art.34453 PMC3366018 22392533

[2] Zhang L Haddouti EM Welle K Burger C Wirtz DC Schildberg FA. The effects of biomaterial implant wear debris on osteoblasts. Front Cell Dev Biol (2020) 3 8:352. doi: 10.3389/fcell.2020.00352 PMC7283386 32582688

[3] Szczesiul J Bielecki M. A review of total hip arthroplasty comparison in FNF and OA patients. Adv Orthop (2021) 2021:1–6. doi: 10.1155/2021/5563500 PMC8463253 34567807

[4] Goodman SB Gibon E Gallo J Takagi M. Macrophage polarization and the osteoimmunology of periprosthetic osteolysis. Curr Osteoporos Rep (2022) 20(1):43–52. doi: 10.1007/s11914-022-00720-3 35133558

[5] Higgs GB Hanzlik JA MacDonald DW Gilbert JL Rimnac CM Kurtz SM. Is increased modularity associated with increased fretting and corrosion damage in metal-on-metal total hip arthroplasty devices? J Arthroplasty (2013) 28(8 0):2–6. doi: 10.1016/j.arth.2013.05.040 23910820 PMC3971476

[6] Scharf B Clement CC Zolla V Perino G Yan B Elci SG. Molecular analysis of chromium and cobalt-related toxicity. Sci Rep (2014) 17 4(1):5729. doi: 10.1038/srep05729 PMC4103093 25034144

[7] Ihn HJ Kim K Cho HS Park EK. Pentamidine inhibits titanium particle-induced osteolysis in vivo and receptor activator of nuclear factor-κB ligand-mediated osteoclast differentiation *in vitro* . Tissue Eng Regener Med (2019) 16(3):265–73. doi: 10.1007/s13770-019-00186-y PMC6542890 31205855

[8] Bechtel CP Gebhart JJ Tatro JM Kiss-Toth E Wilkinson JM. Greenfield EM. Particle-induced osteolysis is mediated by TIRAP/mal in vitro and in vivo: dependence on adherent pathogen-associated molecular patterns. J Bone Joint Surgery. (2016) 98(4):285–94. doi: 10.2106/JBJS.O.00736 26888676

[9] Vaculova J Gallo J Hurnik P Motyka O Goodman SB Dvorackova J. Low intrapatient variability of histomorphological findings in periprosthetic tissues from revised metal/ceramic on polyethylene joint arthroplasties: Intra-patient tissue response to TJA has similar appearance regardless of site. J BioMed Mater Res (2018) 106(5):2008–18. doi: 10.1002/jbm.b.33990 29044940

[10] Fritz J Lurie B Miller TT Potter HG. MR imaging of hip arthroplasty implants. RadioGraphics. > (2014) 34(4):E106–32. doi: 10.1148/rg.344140010 25019450

[11] Fritz J Lurie B Potter HG. MR imaging of knee arthroplasty implants. RadioGraphics. > (2015) 35(5):1483–501. doi: 10.1148/rg.2015140216 PMC4613886 26295591

[12] Marshall A Ries MD Paprosky W. How prevalent are implant wear and osteolysis, and how has the scope of osteolysis changed since 2000. J Am Acad Orthopaedic Surgeons (2008) 16:S1–6. doi: 10.5435/00124635-200800001-00003 18612002

[13] Jonitz-Heincke S Sellin M-L Seyfarth P Peters K Mueller-Hilke B Fiedler T. Analysis of cellular activity short-term exposure to cobalt and chromium ions in mature human osteoblasts. Materials. > (2019) 12(17):2771. doi: 10.3390/ma12172771 31466377 PMC6747798

[14] Lohberger B Eck N Glaenzer D Lichtenegger H Ploszczanski L Leithner A. Cobalt chromium molybdenum surface modifications alter the osteogenic differentiation potential of human mesenchymal stem cells. Materials. > (2020) 13(19):4292. doi: 10.3390/ma13194292 32992906 PMC7579014

[15] Krenn V Morawietz L Perino G Kienapfel H Ascherl R Hassenpflug GJ. Revised histopathological consensus classification of joint implant related pathology. Pathol - Res Practice. (2014) 210(12):779–86. doi: 10.1016/j.prp.2014.09.017 25454771

[16] Lachiewicz PF Kleeman LT Seyler T. Bearing surfaces for total hip arthroplasty. J Am Acad Orthopaedic Surgeons (2018) 26(2):45–57. doi: 10.5435/JAAOS-D-15-00754 29303922

[17] Mitchell RN Schoen FJ. Functional tissue architecture, homeostasis, and responses to injury(2020). Elsevier. Available at: https://linkinghub.elsevier.com/retrieve/pii/B9780128161371000441 (Accessed 2023 Apr 17).

[18] Eming SA Martin P Tomic-Canic M. Wound repair and regeneration: Mechanisms, signaling, and translation. Sci Transl Med (2014) 6(265). doi: 10.1126/scitranslmed.3009337 PMC4973620 25473038

[19] Babensee JE. Inflammation, wound healing, the foreign-body response, and alternative tissue responses, in: Biomaterials science (2020). Elsevier. Available at: http://journals.lww.com/00004669-199701000-00009 (Accessed 2023 Apr 17).

[20] Rodriguez A Meyerson H Anderson JM. Quantitative*in vivo* cytokine analysis at synthetic biomaterial implant sites(2008) (Accessed 2023 Apr 17). 10.1002/jbm.a.31939 PMC3864694 18431759

[21] Nilsson B Ekdahl KN Mollnes TE Lambris JD. The role of complement in biomaterial-induced inflammation. Mol Immunol (2007) 44(1–3):82–94. doi: 10.1016/j.molimm.2006.06.020 16905192

[22] Konttinen YT Pajarinen J Takakubo Y Gallo J Nich C Takagi M. Macrophage polarization and activation in response to implant debris: influence by “Particle disease” and “Ion disease. ” J Long Term Eff Med Implants (2014) 24(4):267–81. doi: 10.1615/JLongTermEffMedImplants.2014011355 PMC4373605 25747030

[23] Rao AJ Gibon E Ma T Yao Z Smith RL Goodman SB. Revision joint replacement, wear particles, and macrophage polarization. Acta Biomaterialia. (2012) 8(7):2815–23. doi: 10.1016/j.actbio.2012.03.042 PMC3730834 22484696

[24] Vasconcelos DM Ribeiro-da-Silva M Mateus A Alves CJ MaChado GC MaChado-Santos J. Immune response and innervation signatures in aseptic hip implant loosening. J Transl Med (2016) 14(1):205. doi: 10.1186/s12967-016-0950-5 27387445 PMC4937545

[25] Colombi A Schena D Castelli CC. Total hip arthroplasty planning. EFORT Open Rev (2019) 4(11):626–32. doi: 10.1302/2058-5241.4.180075 PMC6851526 31754469

[26] Teeter MG Goyal P Yuan X Howard JL Lanting BA. Change in acetabular cup orientation from supine to standing position and its effect on wear of highly crosslinked polyethylene. J Arthroplasty. (2018) 33(1):263–7. doi: 10.1016/j.arth.2017.08.016 28917617

[27] Rasquinha VJ Ranawat CS Cervieri CL Rodriguez JA. The press-fit condylar modular total knee system with a posterior cruciate-substituting design. JBJS ORG (2006) 88-A(5):1006–10. doi: 10.2106/JBJS.C.01104 16651575

[28] Sharkey PF Lichstein PM Shen C Tokarski AT Parvizi J. Why are total knee arthroplasties failing today–has anything changed after 10 years? J Arthroplasty (2014) 29(9):1774–8. doi: 10.1016/j.arth.2013.07.024 25007726

[29] Corvec S Portillo ME Pasticci BM Borens O Trampuz A. Epidemiology and new developments in the diagnosis of prosthetic joint infection. Int J Artif Organs. (2012) 35(10):923–34. doi: 10.5301/ijao.5000168 23138706

[30] Zimmerli W. Prosthetic-joint infections. New Engl J Med (2004) 351:1645–54. doi: 10.1056/NEJMra040181 15483283

[31] Bach CM Sturmer R Nogler M Wimmer C Biedermann R Krismer M. Total knee arthroplasty infection: Significance of delayed aspiration. J Arthroplasty. (2002) 17(5):615–8. doi: 10.1054/arth.2002.32140 12168179

[32] Kaplan SS Heine RP Simmons RL. Defensins impair phagocytic killing by neutrophils in biomaterial-related infection. Infect Immun (1999) 67(4):1640–5. doi: 10.1128/IAI.67.4.1640-1645.1999 PMC96507 10084997

[33] Hazelwood KJ O’Rourke M Stamos VP McMillan RD Beigler D Robb WJ. Case series report: Early cement–implant interface fixation failure in total knee replacement. Knee. (2015) 22(5):424–8. doi: 10.1016/j.knee.2015.02.016 25795544

[34] Cheng K Pruitt L Zaloudek C Ries MD. Osteolysis caused by tibial component debonding in total knee arthroplasty. Clin Orthopaedics Related Res (2006) 443:333–6. doi: 10.1097/01.blo.0000196044.42413.c7 16462459

[35] Foran JRH Whited BW Sporer SM. Early aseptic loosening with a precoated low-profile tibial component. J Arthroplasty. (2011) 26(8):1445–50. doi: 10.1016/j.arth.2010.11.002 21236628

[36] Seeger JB Jaeger S Bitsch RG Mohr G Röhner E Clarius M. The effect of bone lavage on femoral cement penetration and interface temperature during oxford unicompartmental knee arthroplasty with cement. J Bone Joint Surgery. (2013) 95(1):48–53. doi: 10.2106/JBJS.K.01116 23283372

[37] Arsoy D Pagnano MW Lewallen DG Hanssen AD Sierra RJ. Aseptic tibial debonding as a cause of early failure in a modern total knee arthroplasty design. Clin Orthopaedics Related Res (2013) 471(1):94–101. doi: 10.1007/s11999-012-2467-4 PMC3528903 22790529

[38] Goodman SB Gallo J. Periprosthetic osteolysis: mechanisms, prevention and treatment. JCM. > (2019) 8(12):2091. doi: 10.3390/jcm8122091 31805704 PMC6947309

[39] Hodges NA Sussman EM Stegemann JP. Aseptic and septic prosthetic joint loosening: Impact of biomaterial wear on immune cell function, inflammation, and infection. Biomaterials. > (2021) 278:121127. doi: 10.1016/j.biomaterials.2021.121127 34564034

[40] Schroer WC Berend KR Lombardi AV Barnes CL Bolognesi MP Berend ME. Why are total knees failing today? Etiology of total knee revision in 2010 and 2011. J Arthroplasty. (2013) 28(8):116–9. doi: 10.1016/j.arth.2013.04.056 23954423

[41] Feng X Gu J Zhou Y. Primary total hip arthroplasty failure: aseptic loosening remains the most common cause of revision. Am J Transl Res (2022) 14(10):7080–9. PMC9641425 36398241

[42] Katz JN Wright J Wright EA Losina E. Failures of total hip replacement: A population-based perspective. Orthopaedic Journal at Harvard Medical School 9:101–6.

[43] Catelas I Petit A Zukor DJ Antoniou J Huk OL. TNF-α secretion and macrophage mortality induced by cobalt and chromium ions in *vitro*-Qualitative analysis of apoptosis. Biomaterials. > (2003) 24(3):383–91. doi: 10.1016/S0142-9612(02)00351-4 12423593

[44] Granchi D Amato I Battistelli L Ciapetti G Pagani S Avnet S. Molecular basis of osteoclastogenesis induced by osteoblasts exposed to wear particles. Biomaterials. > (2005) 26(15):2371–9. doi: 10.1016/j.biomaterials.2004.07.045 15585240

[45] Ingham E Green TR Stone MH Kowalski R Watkins N Fisher J. Production of TNF-α and bone resorbing activity by macrophages in response to different types of bone cement particles. Biomaterials. > (2000) 21(10):1005–13. doi: 10.1016/S0142-9612(99)00261-6 10768752

[46] Brown C Fisher J Ingham E. Biological effects of clinically relevant wear particles from metal-on-metal hip prostheses. Proc Inst Mech Eng H. (2006) 220(2):355–69. doi: 10.1243/095441105X63291 16669401

[47] Mahendra G Pandit H Kliskey K Murray D Gill HS Athanasou N. Necrotic and inflammatory changes in metal-on-metal resurfacing hip arthroplasties: Relation to implant failure and pseudotumor formation. Acta Orthopaedica. (2009) 80(6):653–9. doi: 10.3109/17453670903473016 PMC2823316 19995315

[48] Gibon E Córdova LA Lu L Lin TH Yao Z Hamadouche M. The biological response to orthopedic implants for joint replacement. II: Polyethylene, ceramics, PMMA, and the foreign body reaction. J BioMed Mater Res (2017) 105(6):1685–91. doi: 10.1002/jbm.b.33676 PMC5536115 27080740

[49] Perino G Sunitsch S Huber M Ramirez D Gallo J Vaculova J. Diagnostic guidelines for the histological particle algorithm in the periprosthetic neo-synovial tissue. BMC Clin Pathol (2018) 18(1):7. doi: 10.1186/s12907-018-0074-3 30158837 PMC6109269

[50] Williams JA. Wear and wear particles—some fundamentals. Tribology Int (2005) 38(10):863–70. doi: 10.1016/j.triboint.2005.03.007

[51] McKellop HA Hart A Park SH Hothi H Campbell P Skinner JA. A lexicon for wear of metal-on-metal hip prostheses. J Orthop Res (2014) 32(9):1221–33. doi: 10.1002/jor.22651 24844814

[52] Hall DJ Pourzal R Lundberg HJ Mathew MT Jacobs JJ Urban RM. Mechanical, chemical and biological damage modes within head-neck tapers of CoCrMo and Ti6Al4V contemporary hip replacements. J BioMed Mater Res (2018) 106(5):1672–85. doi: 10.1002/jbm.b.33972 PMC5826871 28842959

[53] Caicedo MS Samelko L McAllister K Jacobs JJ Hallab NJ. Increasing both CoCrMo-alloy particle size and surface irregularity induces increased macrophage inflammasome activation in *vitro* potentially through lysosomal destabilization mechanisms. J Orthop Res (2013) 31(10):1633–42. doi: 10.1002/jor.22411 PMC4028039 23794526

[54] Knutsen AR Lau N Longjohn DB Ebramzadeh E Sangiorgio SN. Periprosthetic femoral bone loss in total hip arthroplasty: systematic analysis of the effect of stem design. HIP Int (2017) 27(1):26–34. doi: 10.5301/hipint.5000413 27515762

[55] Cristofolini L. Critical examination of stress shielding evaluation of hip prostheses. Crit Rev BioMed Eng (2017) 45(1–6):549–623. doi: 10.1615/CritRevBiomedEng.v45.i1-6.190 29953388

[56] Linde KN Rytter S Søballe K Madsen F Langdahl B Stilling M. Component migration, bone mineral density changes, and bone turnover markers in cementless and cemented total knee arthroplasty: a prospective randomized RSA study in 53 patients with 2-year follow-up. Knee Surg Sports Traumatol Arthrosc. (2022) 30(9):3100–13. doi: 10.1007/s00167-022-06860-4 35099597

[57] Konttinen YT Zhao D Beklen A Ma G Takagi M Kivelä-Rajamäki M. The microenvironment around total hip replacement prostheses. Clin Orthopaedics Related Res (2005) 430:28–38. doi: 10.1097/01.blo.0000150451.50452.da 15662301

[58] Amirhosseini M Andersson G Aspenberg P Fahlgren A. Mechanical instability and titanium particles induce similar transcriptomic changes in a rat model for periprosthetic osteolysis and aseptic loosening. Bone Rep (2017) 7:17–25. doi: 10.1016/j.bonr.2017.07.003 28795083 PMC5544474

[59] Bratengeier C Bakker AD Fahlgren A. Mechanical loading releases osteoclastogenesis-modulating factors through stimulation of the P2X7 receptor in hematopoietic progenitor cells. J Cell Physiol (2019) 234(8):13057–67. doi: 10.1002/jcp.27976 PMC13483287 30536959

[60] Fahlgren A Bratengeier C Semeins CM Klein-Nulend J Bakker AD. Supraphysiological loading induces osteocyte-mediated osteoclastogenesis in a novel in *vitro* model for bone implant loosening. J Orthop Res (2018) 36(5):1425–34. doi: 10.1002/jor.23780 29068483

[61] Madsen RV Nam D Schilcher J Dvorzhinskiy A Sutherland JP Bostrom FM. Mechanical instability induces osteoclast differentiation independent of the presence of a fibrous tissue interface and osteocyte apoptosis in a rat model for aseptic loosening. Acta Orthopaedica. (2020) 91(1):115–20. doi: 10.1080/17453674.2019.1695351 PMC7006729 31762353

[62] Mjöberg B. Does particle disease really exist? Acta Orthop (2018) 89(1):130–2. doi: 10.1080/17453674.2017.1373491 PMC5810822 28914108

[63] Mjöberg B. Hip prosthetic loosening and periprosthetic osteolysis: A commentary. WJO. > (2022) 13(6):574–7. doi: 10.5312/wjo.v13.i6.574 PMC9244959 35949708

[64] Andreev D Liu M Weidner D Kachler K Faas M Grüneboom A. Osteocyte necrosis triggers osteoclast-mediated bone loss through macrophage-inducible C-type lectin. J Clin Invest (2020) 130(9):4811–30. doi: 10.1172/JCI134214 PMC7456234 32773408

[65] Fahlgren A Bostrom MP Yang X Johansson L Edlund U Agholme F. Fluid pressure and flow as a cause of bone resorption. Acta Orthopaedica. (2010) 81(4):508–16. doi: 10.3109/17453674.2010.504610 PMC2917576 20718695

[66] Mann KA Miller MA Rossow JK Tatusko ME Horton JA Damron TA. Progressive loss of implant fixation in a preclinical rat model of cemented knee arthroplasty. J Orthop Res (2021) 39(11):2353–62. doi: 10.1002/jor.24977 PMC8243390 33382095

[67] Miller MA Hardy WR Oest ME Mann KA. Potential for supraphysiologic fluid shear stresses in a rat cemented knee replacement model. J Orthopaedic Res (2023) 41(1):94–103. doi: 10.1002/jor.25326 PMC9509496 35332943

[68] Alidousti H Taylor M Bressloff NW. Do capsular pressure and implant motion interact to cause high pressure in the periprosthetic bone in total hip replacement? J Biomechanical Eng (2011) 133(12):121001. doi: 10.1115/1.4005455 22206418

[69] Miller MA Terbush MJ Goodheart JR Izant TH Mann KA. Increased initial cement–bone interlock correlates with reduced total knee arthroplasty micro-motion following in *vivo* service. J Biomech. (2014) 47(10):2460–6. doi: 10.1016/j.jbiomech.2014.04.016 PMC4100248 24795171

[70] Mann KA Miller MA. Fluid–structure interactions in micro-interlocked regions of the cement–bone interface. Comput Methods Biomechanics Biomed Engineering. (2014) 17(16):1809–20. doi: 10.1080/10255842.2013.767336 PMC3686830 23480611

[71] Jandl NM Rolvien T Gätjen D Jonitz-Heincke A Springer A Krenn V. Recurrent arthrocele and sterile sinus tract formation due to ceramic wear as a differential diagnosis of periprosthetic joint infection — a case report. Acta Orthopaedica. (2019) 90(5):501–4. doi: 10.1080/17453674.2019.1616997 PMC6746295 31094272

[72] Perino G De Martino I Zhang L Xia Z Gallo J Natu S. The contribution of the histopathological examination to the diagnosis of adverse local tissue reactions in arthroplasty. EFORT Open Rev (2021) 6(6):399–419. doi: 10.1302/2058-5241.6.210013 34267931 PMC8246109

[73] Campbell P Ebramzadeh E Nelson S Takamura K De Smet K Amstutz HC. Histological features of pseudotumor-like tissues from metal-on-metal hips. Clin Orthopaedics Related Res (2010) 468(9):2321–7. doi: 10.1007/s11999-010-1372-y PMC2914255 20458645

[74] Bertrand J Delfosse D Mai V Awiszus F Harnisch K Lohmann CH. Ceramic prosthesis surfaces induce an inflammatory cell response and fibrotic tissue changes. Bone Joint J (2018) 100-B(7):882–90. doi: 10.1302/0301-620X.100B7.BJJ-2017-1590.R2 29954216

[75] Ingham E Fisher J. The role of macrophages in osteolysis of total joint replacement. Biomaterials. > (2005) 26(11):1271–86. doi: 10.1016/j.biomaterials.2004.04.035 15475057

[76] Nich C Takakubo Y Pajarinen J Ainola M Salem A Sillat T. Macrophages-Key cells in the response to wear debris from joint replacements: Macrophage Response to Wear Debris. J BioMed Mater Res (2013) 101(10):3033–45. doi: 10.1002/jbm.a.34599 PMC3775910 23568608

[77] van der Voort P Pijls BG Nieuwenhuijse MJ Jasper J Fiocco M Plevier JWM. Early subsidence of shape-closed hip arthroplasty stems is associated with late revision: A systematic review and meta-analysis of 24 RSA studies and 56 survival studies. Acta Orthopaedica. (2015) 86(5):575–85. doi: 10.3109/17453674.2015.1043832 PMC4564780 25909455

[78] Bosetti M Massè A Navone R Cannas M. Biochemical and histological evaluation of human synovial-like membrane around failed total hip replacement prostheses during in *vitro* mechanical loading. J Mater Sci Mater Med (2001) 12(8):693–8. doi: 10.1023/A:1011216509099 15348240

[79] Goldring SR Schiller AL Roelke M Rourke CM O’Neil DA Harris WH. The synovial-like membrane at the bone-cement interface in loose total hip replacements and its proposed role in bone lysis. J Bone Joint Surg Am (1983) 65(5):575–84. doi: 10.2106/00004623-198365050-00001 6304106

[80] Pajarinen J Gallo J Takagi M Goodman SB Mjöberg B. Letter: Particle disease really does exist. Response: Particle disease, late loosening and Occam’s razor.: An evidence based rebuttal to Dr. Mjöberg’s opinion letter. Acta Orthopaedica. (2018) 89(1):133–6. doi: 10.1080/17453674.2017.1402463 PMC5810823 29143557

[81] Hochman MG Melenevsky YV Metter DF Roberts CC Bencardino JT Cassidy RC. ACR appropriateness criteria ^®^ Imaging after total knee arthroplasty. J Am Coll Radiology. (2017) 14(11):S421–48. doi: 10.1016/j.jacr.2017.08.036 29101982

[82] Mulcahy H Chew FS. Current concepts in knee replacement: complications. Am J Roentgenology (2014) 202(1):W76–86. doi: 10.2214/AJR.13.11308 24370168

[83] Katzer A Lœhr JF. Early loosening of hip replacements: causes, course and diagnosis. J Orthopaed Traumatol. (2003) 4(3):105–16. doi: 10.1007/s10195-003-0021-6

[84] Kosashvili Y Alvi M Mayne IP Safir O Gross A Backstein D. Immediate recovery room radiographs after primary total knee arthroplasty—why do we keep doing them? Int Orthopaedics (SICOT) (2010) 34(8):1167–73. doi: 10.1007/s00264-009-0888-9 PMC2989086 19826813

[85] Glaser D Lotke P. Cost-effectiveness of immediate postoperative radiographs after uncomplicated total knee arthroplasty. J Arthroplasty. (2000) 15(4):475–8. doi: 10.1054/arth.2000.4338 10884208

[86] Hassan S Wall A Ayyawamy B Rogers S Mills S Charalambous C. Is there a need for early post-operative x-rays in primary total knee replacements? Experience of a centre in the UK. annals. > (2012) 94(3):199–200. doi: 10.1308/003588412X13171221501780 PMC3705236 22507727

[87] Busch A Jäger M Beck S Wegner A Portegys E Wassenaar D. Metal artefact reduction sequences (MARS) in magnetic resonance imaging (MRI) after total hip arthroplasty (THA). BMC Musculoskelet Disord (2022) 23:620. doi: 10.1186/s12891-022-05560-x 35764987 PMC9238049

[88] Jonitz-Heincke A Lochner K Schulze C Pohle D Pustlauk W Hansmann D. Contribution of human osteoblasts and macrophages to bone matrix degradation and proinflammatory cytokine release after exposure to abrasive endoprosthetic wear particles. Mol Med Rep (2016) 14(2):1491–500. doi: 10.3892/mmr.2016.5415 PMC4940096 27357630

[89] van Furth R. Cells of the mononuclear phagocyte system, in: Mononuclear phagocytes (1980). Dordrecht: Springer Netherlands (Accessed 2023 Apr 18).

[90] North RJ. The mitotic potential of fixed phagocytes in the liver as revealed during the development of cellular immunity. J Exp Med (1969) 130(2):315–26. doi: 10.1084/jem.130.2.315 PMC2138678 4978534

[91] Merad M Manz MG Karsunky H Wagers A Peters W Charo I. Langerhans cells renew in the skin throughout life under steady-state conditions. Nat Immunol (2002) 3(12):1135–41. doi: 10.1038/ni852 PMC4727838 12415265

[92] Ajami B Bennett JL Krieger C Tetzlaff W Rossi FMV. Local self-renewal can sustain CNS microglia maintenance and function throughout adult life. Nat Neurosci (2007) 10(12):1538–43. doi: 10.1038/nn2014 18026097

[93] Yona S Gordon S. From the reticuloendothelial to mononuclear phagocyte system – the unaccounted years(2015) (Accessed 2023 Apr 18). 10.3389/fimmu.2015.00328 PMC4486871 26191061

[94] Guilliams M Mildner A Yona S. Developmental and functional heterogeneity of monocytes. Immunity. > (2018) 49(4):595–613. doi: 10.1016/j.immuni.2018.10.005 30332628

[95] Yao Y Cai X Ren F Ye Y Wang F Zheng C. The macrophage-osteoclast axis in osteoimmunity and osteo-related diseases. Front Immunol (2021) 12:664871. doi: 10.3389/fimmu.2021.664871 33868316 PMC8044404

[96] Goodman SB. Wear particles, periprosthetic osteolysis and the immune system. Biomaterials. > (2007) 28(34):5044–8. doi: 10.1016/j.biomaterials.2007.06.035 PMC2065897 17645943

[97] Kim KJ Chiba J Rubash HE. *In vivo* and in *vitro* analysis of membranes from hip prostheses inserted without cement. J Bone Joint Surg Am (1994) 76(2):172–80. doi: 10.2106/00004623-199402000-00002 8113250

[98] Nich C Goodman SB. The role of macrophages in the biological reaction to wear debris from joint replacements. J Long Term Eff Med Implants. (2014) 24(4):259–65. doi: 10.1615/JLongTermEffMedImplants.2014010562 PMC4366682 25747029

[99] Baxter RM MacDonald DW Kurtz SM Steinbeck MJ. Characteristics of highly cross-linked polyethylene wear debris in vivo. J BioMed Mater Res (2013) 467–75. doi: 10.1002/jbm.b.32902 PMC3928672 23436587

[100] Fisher J Bell J Barbour PSM Tipper JL Mattews JB Besong AA. A novel method for the prediction of functional biological activity of polyethylene wear debris. Proc Inst Mech Eng H. (2001) 215(2):127–32. doi: 10.1243/0954411011533599 11382071

[101] Natu S Sidaginamale RP Gandhi J Langton DJ Nargol AVF. Adverse reactions to metal debris: histopathological features of periprosthetic soft tissue reactions seen in association with failed metal on metal hip arthroplasties. J Clin Pathol (2012) 65(5):409–18. doi: 10.1136/jclinpath-2011-200398 22422805

[102] Ma TL Chen JX Ke ZR Zhu P Hu YH Xie J. Targeting regulation of stem cell exosomes: Exploring novel strategies for aseptic loosening of joint prosthesis. Front Bioeng Biotechnol (2022) 10:925841. doi: 10.3389/fbioe.2022.925841 36032702 PMC9399432

[103] Takagi M Tamaki Y Hasegawa H Takakubo Y Konttinen L Tiainen VM. Toll-like receptors in the interface membrane around loosening total hip replacement implants. J Biomed Materials Res Part A (2007) 81A(4):1017–26. doi: 10.1002/jbm.a.31235 17415764

[104] Hu Y Wang Y Chen T Hao Z Cai L Li J. Exosome: function and application in inflammatory bone diseases. Oxid Med Cell Longev (2021) 2021:1–17. doi: 10.1155/2021/6324912 PMC8423581 34504641

[105] Dyskova T Gallo J Kriegova E. The role of the chemokine system in tissue response to prosthetic by-products leading to periprosthetic osteolysis and aseptic loosening. Front Immunol (2017) 8:1026. doi: 10.3389/fimmu.2017.01026 28883822 PMC5573717

[106] Zhao B. Does TNF promote or restrain osteoclastogenesis and inflammatory bone resorption? Crit Rev Immunol (2018) 38(4):253–61. doi: 10.1615/CritRevImmunol.2018025874 PMC6422676 30806242

[107] Adamopoulos IE. Inflammation in bone physiology and pathology. Curr Opin Rheumatol (2018) 30(1):59–64. doi: 10.1097/BOR.0000000000000449 29016371 PMC5963529

[108] Alippe Y Mbalaviele G. Omnipresence of inflammasome activities in inflammatory bone diseases. Semin Immunopathol (2019) 41(5):607–18. doi: 10.1007/s00281-019-00753-4 PMC6814643 31520179

[109] Konttinen YT Pajarinen J. Adverse reactions to metal-on-metal implants. Nat Rev Rheumatol (2013) 9(1):5–6. doi: 10.1038/nrrheum.2012.218 23208186

[110] Fort BP Dubyak GR Greenfield EM. Lysosomal disruption by orthopedic wear particles induces activation of the NLRP3 inflammasome and macrophage cell death by distinct mechanisms. J Orthop Res (2021) 39(3):493–505. doi: 10.1002/jor.24826 32779803 PMC8201664

[111] Jämsen E Pajarinen J Kouri VP Rahikkala A Goodman SB Manninen M. Tumor necrosis factor primes and metal particles activate the NLRP3 inflammasome in human primary macrophages. Acta Biomaterialia. (2020) 108:347–57. doi: 10.1016/j.actbio.2020.03.017 PMC7729209 32194260

[112] Ricciardi BF Nocon AA Jerabek SA Wilner G Kaplowitz E Goldring SR. Histopathological characterization of corrosion product associated adverse local tissue reaction in hip implants: a study of 285 cases. BMC Clin Pathol (2016) 16(1):3. doi: 10.1186/s12907-016-0025-9 26924942 PMC4769839

[113] Kwon YM Ostlere SJ McLardy-Smith P Athanasou NA Gill HS Murray DW. “Asymptomatic” Pseudotumors after metal-on-metal hip resurfacing arthroplasty: prevalence and metal ion study. J Arthroplasty. (2011) 26(4):511–8. doi: 10.1016/j.arth.2010.05.030 20591612

[114] Bauer TW Schils J. The pathology of total joint arthroplasty. Skeletal Radiol (1999) 28(9):483–97. doi: 10.1007/s002560050552 10525792

[115] Agidigbi TS Kim C. Reactive oxygen species in osteoclast differentiation and possible pharmaceutical targets of ROS-mediated osteoclast diseases. IJMS. > (2019) 20(14):3576. doi: 10.3390/ijms20143576 31336616 PMC6678498

[116] Nascimento Da Conceicao V Sun Y Ramachandran K Chauhan A Raveendran A Venkatesan M. Resolving macrophage polarization through distinct Ca2+ entry channel that maintains intracellular signaling and mitochondrial bioenergetics. iScience. > (2021) 24(11):103339. doi: 10.1016/j.isci.2021.103339 34816101 PMC8591423

[117] Deschaseaux F Sensébé L Heymann D. Mechanisms of bone repair and regeneration. Trends Mol Med (2009) 15(9):417–29. doi: 10.1016/j.molmed.2009.07.002 19740701

[118] Landgraeber S von Knoch M Löer F Brankamp J Tsokos M Grabellus F. Association between apoptotis and CD4 ^+^/CD8 ^+^ T-lymphocyte ratio in aseptic loosening after total hip replacement. Int J Biol Sci (2009) 5:182–91. doi: 10.7150/ijbs.5.182 PMC2640493 19214244

[119] Brooks PJ Glogauer M McCulloch CA. An overview of the derivation and function of multinucleated giant cells and their role in pathologic processes. Am J Pathology. (2019) 189(6):1145–58. doi: 10.1016/j.ajpath.2019.02.006 30926333

[120] Anderson JM Defife K Mcnally A Collier T Jenney C. Monocyte, macrophage and foreign body giant cell interactions with molecularly engineered surfaces. J Materials Science: Materials Med (1999) 10(10):579–88. doi: 10.1023/A:1008976531592 15347970

[121] Helming L Gordon S. Macrophage fusion induced by IL-4 alternative activation is a multistage process involving multiple target molecules. Eur J Immunol (2007) 37(1):33–42. doi: 10.1002/eji.200636788 17154265

[122] DeFife KM Jenney CR McNally AK Colton E Anderson JM. Interleukin-13 induces human monocyte/macrophage fusion and macrophage mannose receptor expression. J Immunol (1997) 158(7):3385–90. doi: 10.4049/jimmunol.158.7.3385 9120298

[123] McNally AK Anderson JM. Foreign body-type multinucleated giant cell formation is potently induced by α-tocopherol and prevented by the diacylglycerol kinase inhibitor R59022. Am J Pathol (2003) 163(3):1147–56. doi: 10.1016/S0002-9440(10)63474-8 PMC1868253 12937156

[124] Moreno JL Mikhailenko I Tondravi MM Keegan AD. IL-4 promotes the formation of multinucleated giant cells from macrophage precursors by a STAT6-dependent, homotypic mechanism: contribution of E-cadherin. J Leukocyte Biol (2007) 82(6):1542–53. doi: 10.1189/jlb.0107058 17855502

[125] Rayner KJ. Cell death in the vessel wall: The good, the bad, the ugly. Arterioscler Thromb Vasc Biol (2017) 37(7):e75–81. doi: 10.1161/ATVBAHA.117.309229 PMC5584709 28637702

[126] Konttinen YT Takagi M Mandelin J Lassus J Salo J Ainola M. Acid attack and cathepsin K in bone resorption around total hip replacement prosthesis. J Bone Miner Res (2001) 16(10):1780–6. doi: 10.1359/jbmr.2001.16.10.1780 11585341

[127] Ma GF Ali A Verzijl N Hanemaaijer R TeKoppele J T. Konttinen Y. Increased collagen degradation around loosened total hip replacement implants. Arthritis Rheumatism. (2006) 54(9):2928–33. doi: 10.1002/art.22064 16948130

[128] Perino G Ricciardi BF Jerabek SA Martignoni G Wilner G Maass D. Implant based differences in adverse local tissue reaction in failed total hip arthroplasties: a morphological and immunohistochemical study. BMC Clin Pathol (2014) 14:39. doi: 10.1186/1472-6890-14-39 25242891 PMC4169255

[129] Mestres G Carter SSD Hailer NP Diez-Escudero A. A practical guide for evaluating the osteoimmunomodulatory properties of biomaterials. Acta Biomaterialia. (2021) 130:115–37. doi: 10.1016/j.actbio.2021.05.038 34087437

[130] Lorenzo J. Cytokines and bone: osteoimmunology, in: Bone regulators and osteoporosis therapy (2020). Cham: Springer International Publishing (Accessed 2023 Apr 26). Handbook of Experimental Pharmacology; vol. 262. 10.1007/164_2019_346 32006259

[131] Kim YG Lee CK Nah SS Mun SH Yoo B Moon HB. Human CD4+CD25+ regulatory T cells inhibit the differentiation of osteoclasts from peripheral blood mononuclear cells. Biochem Biophys Res Commun (2007) 357(4):1046–52. doi: 10.1016/j.bbrc.2007.04.042 17462597

[132] Wu K Yu B Li D Tian Y Liu Y Jiang J. Recent advances in nanoplatforms for the treatment of osteosarcoma. Front Oncol (2022) 12:805978. doi: 10.3389/fonc.2022.805978 35242707 PMC8885548

[133] Niki Y Matsumoto H Otani T Yatabe T Kondo M Yoshimine F. Screening for symptomatic metal sensitivity: a prospective study of 92 patients undergoing total knee arthroplasty. Biomaterials. > (2005) 26(9):1019–26. doi: 10.1016/j.biomaterials.2004.03.038 15369690

[134] Carossino AM Carulli C Ciuffi S Carossino R Zappoli Thyrion GD Zonefrati R. Hypersensitivity reactions to metal implants: laboratory options. BMC Musculoskelet Disord (2016) 17(1):486. doi: 10.1186/s12891-016-1342-y 27881114 PMC5120482

[135] Landgraeber S von Knoch M Löer F Wegner A Tsokos M Hußmann B. Extrinsic and intrinsic pathways of apoptosis in aseptic loosening after total hip replacement. Biomaterials. > (2008) 29(24–25):3444–50. doi: 10.1016/j.biomaterials.2008.04.044 18490052

[136] Steinbeck MJ Jablonowski LJ Parvizi J Freeman TA. The role of oxidative stress in aseptic loosening of total hip arthroplasties. J Arthroplasty. (2014) 29(4):843–9. doi: 10.1016/j.arth.2013.09.001 PMC3965616 24290740

[137] Kusumbe AP Ramasamy SK Adams RH. Coupling of angiogenesis and osteogenesis by a specific vessel subtype in bone. Nature. > (2014) 507(7492):323–8. doi: 10.1038/nature13145 PMC4943525 24646994

[138] Kumar N Saraber P Ding Z Kusumbe AP. Diversity of vascular niches in bones and joints during homeostasis, ageing, and diseases. Front Immunol (2021) 12:798211. doi: 10.3389/fimmu.2021.798211 34975909 PMC8718446

[139] Romeo SG Alawi KM Rodrigues J Singh A Kusumbe AP Ramasamy SK. Endothelial proteolytic activity and interaction with non-resorbing osteoclasts mediate bone elongation. Nat Cell Biol (2019) 21(4):430–41. doi: 10.1038/s41556-019-0304-7 30936475

[140] Jacobs JJ Urban RM Wall J Black J Reid JD Veneman L. Unusual foreign-body reaction to a failed total knee replacement: simulation of a sarcoma clinically and a sarcoid histologically. A Case Rep JBJS. (1995) 77(3):444. doi: 10.2106/00004623-199503000-00015 7890794

[141] Balbouzis T Georgiadis T Grigoris P. Granulomatous lung disease: A novel complication following metallosis from hip arthroplasty. Hip Pelvis. (2016) 28(4):249–53. doi: 10.5371/hp.2016.28.4.249 PMC5240319 28097115

[142] Yang G Tang K Qiao L Li Y Sun S. Identification of Critical Genes and lncRNAs in Osteolysis after Total Hip Arthroplasty and Osteoarthritis by RNA Sequencing. Xu D editor. BioMed Res Int (2021) 2021:1–13. doi: 10.1155/2021/6681925 PMC7984875 33791375

[143] Singh G Reichard T Hameister R Awiszus F Schenk K Feuerstein B. Ballooning osteolysis in 71 failed total ankle arthroplasties. Acta Orthop (2016) 87(4):401–5. doi: 10.1080/17453674.2016.1188346 PMC4967284 27196532

[144] Bos I Zagorski M Boos C Krüger S. Histopathologische Diagnostik der infektiösen Gelenkendoprothesenlockerung. Pathologe. > (2008) 29(4):280–6. doi: 10.1007/s00292-007-0921-y 17639398

[145] Mittal S Revell M Barone F Hardie DL Matharu GS Davenport AJ. Lymphoid aggregates that resemble tertiary lymphoid organs define a specific pathological subset in metal-on-metal hip replacements. Matloubian M editor. PloS One (2013) 8(5):e63470. doi: 10.1371/journal.pone.0063470 PMC3665779 23723985

[146] Qiu J Beckman MJ Qian J Jiranek W. Simultaneous labeling of mast cell proteases and protease mRNAs at the bone–implant interface of aseptically loosened hip implants. J Orthopaedic Res (2005) 23(4):942–8. doi: 10.1016/j.orthres.2005.04.008 15972256

[147] Morawietz L Th G RA Claßen Barden B Otto M Hansen T. Vorschlag für eine Konsensus-Klassifikation der periprothetischen Membran gelockerter Hüft- und Knieendoprothesen. Pathologe (2004) 25(5):265sr6. doi: 10.1007/s00292-004-0710-9 15257415

[148] Vaculová J Hurník P Gallo J Žiak D Motyka O. Immunohistochemical detection of mast and dendritic cells in periprosthetic tissues of aseptically loosened total prostheses. Acta Chir Orthop Traumatol Cech. (2018) 85(5):351–8. doi: 10.55095/achot2018/060 30383532

[149] Rousselle AV Heymann D. Osteoclastic acidification pathways during bone resorption. Bone. > (2002) 30(4):533–40. doi: 10.1016/S8756-3282(02)00672-5 11934642

[150] Koreny T Tunyogi-Csapó M Gál I Vermes C Jacobs JJ Glant TT. The role of fibroblasts and fibroblast-derived factors in periprosthetic osteolysis. Arthritis Rheumatol (2006) 54(10):3221–32. doi: 10.1002/art.22134 17009257

[151] Hartmann ES Köhler MI Huber F Redeker JI Schmitt B Schmitt-Sody M. Factors regulating bone remodeling processes in aseptic implant loosening. J Orthopaedic Res (2017) 35(2):248–57. doi: 10.1002/jor.23274 27116254

[152] Córdova LA Trichet V Escriou V Rosset P Amiaud J Battaglia S. Inhibition of osteolysis and increase of bone formation after local administration of siRNA-targeting RANK in a polyethylene particle-induced osteolysis model. Acta Biomaterialia. (2015) 13:150–8. doi: 10.1016/j.actbio.2014.10.042 25462844

[153] Ostlere S. How to image metal-on-metal prostheses and their complications. Am J Roentgenology. (2011) 197(3):558–67. doi: 10.2214/AJR.11.6840 21862797

[154] Wang Z Liu N Liu K Zhou G Gan J Wang Z. Autophagy mediated CoCrMo particle-induced peri-implant osteolysis by promoting osteoblast apoptosis. Autophagy. > (2015) 11(12):2358–69. doi: 10.1080/15548627.2015.1106779 PMC4835204 26566231

[155] Kang C Wei L Song B Chen L Liu J Deng B. Involvement of autophagy in tantalum nanoparticle-induced osteoblast proliferation. Int J Nanomedicine. (2017) 12:4323–33. doi: 10.2147/IJN.S136281 PMC5473603 28652735

[156] Zhao F Cang D Zhang J Zheng L. Chemerin/ChemR23 signaling mediates the effects of ultra-high molecular weight polyethylene wear particles on the balance between osteoblast and osteoclast differentiation. Ann Transl Med (2021) 9(14):1149. doi: 10.21037/atm-21-2945 34430590 PMC8350637

[157] Philbrick KA Wong CP Kahler-Quesada AM Olson DA Branscum AJ Turner RT. Polyethylene particles inserted over calvarium induce cancellous bone loss in femur in female mice. Bone Rep (2018) 9:84–92. doi: 10.1016/j.bonr.2018.07.001 30094298 PMC6073052

[158] Syggelos SA Aletras AJ Smirlaki I Skandalis SS. Extracellular matrix degradation and tissue remodeling in periprosthetic loosening and osteolysis: focus on matrix metalloproteinases, their endogenous tissue inhibitors, and the proteasome. BioMed Res Int (2013) 2013:230805. doi: 10.1155/2013/230805 23862137 PMC3703793

[159] Massaccesi L Ragone V Papini N Goi G Corsi Romanelli MM Galliera E. Effects of vitamin E-stabilized ultra high molecular weight polyethylene on oxidative stress response and osteoimmunological response in human osteoblast. Front Endocrinol (Lausanne). (2019) 10:203. doi: 10.3389/fendo.2019.00203 31001202 PMC6457167

[160] Galliera E Ragone V Marazzi MG Selmin F Banci L Corsi Romanelli MM. Vitamin E-stabilized UHMWPE: Biological response on human osteoblasts to wear debris. Clinica Chimica Acta (2018) 486:18–25. doi: 10.1016/j.cca.2018.07.012 30006289

